# Supplementary figures and images for: Trichomonas vaginalis extracellular vesicles activate the NLRP3 inflammasome and TLR3-mediated inflammatory cascades in host cells
Source: PLoS Pathog. 2025 Jun 2;21(6):e1013216. doi: 10.1371/journal.ppat.1013216 (PMC12157993; doi:10.1371/journal.ppat.1013216)

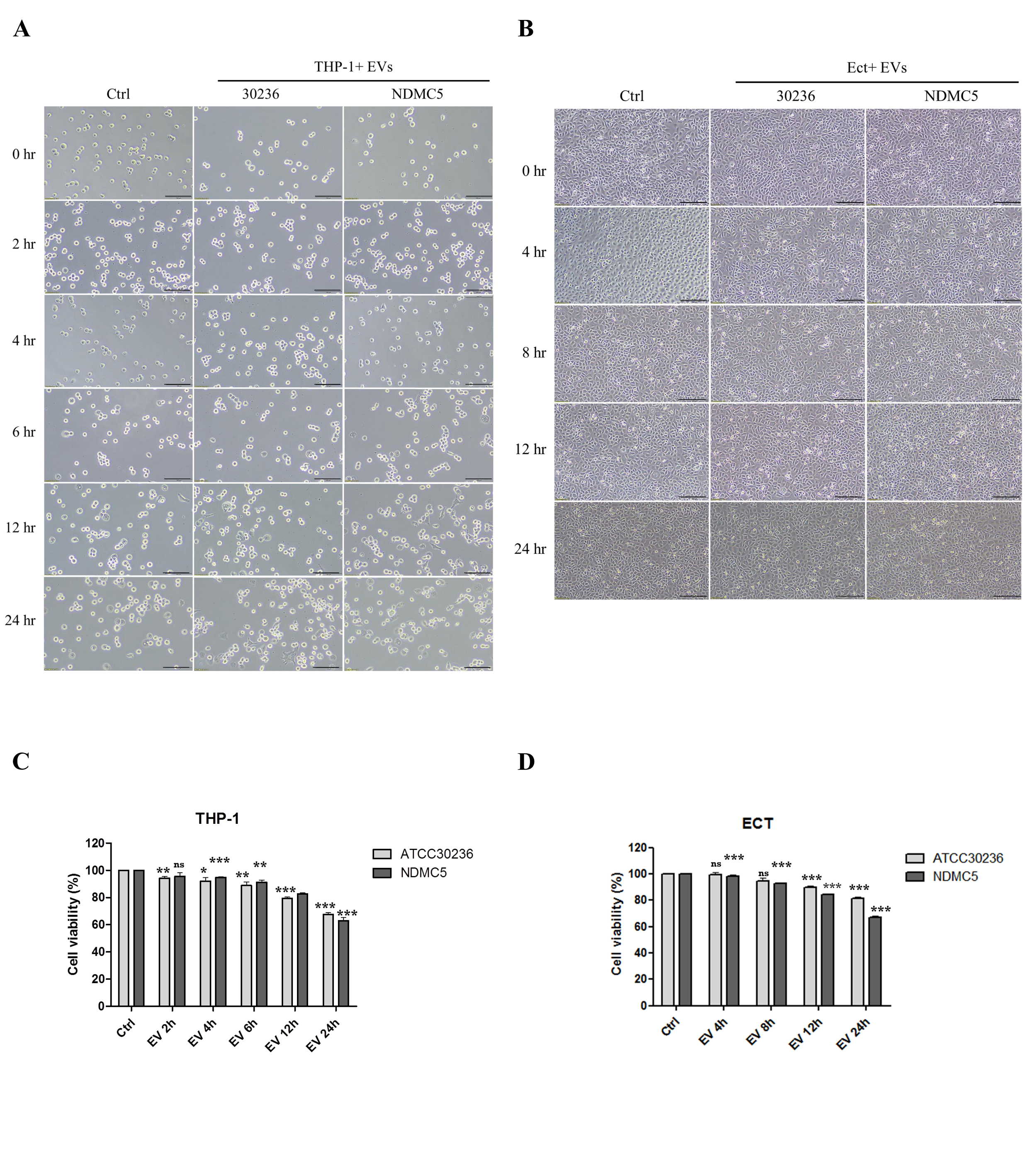

Supplement: S1 Fig — The morphological changes in (A) THP-1 macrophages (2 × 10^5^ cells/ml) and (B) Ect (2 × 10^5^ cells/ml) treated with TV-EVs (30 µg/ml) isolated from the cell line (ATCC 30236) and the clinical strain (NDMC5) for different time intervals were observed. (C) THP-1 macrophages (2 × 10^5^ cells/ml) or (D) Ect (2 × 10^5^ cells/ml) were co-cultured with TV-EVs (30 µg/ml) purified from different strains for different time intervals, and the cell viability was determined using the CCK-8 assay. Scale bar: 1µm. **p < 0.01, ***p < 0.001. (TIFF) [file ppat.1013216.s001.tiff]

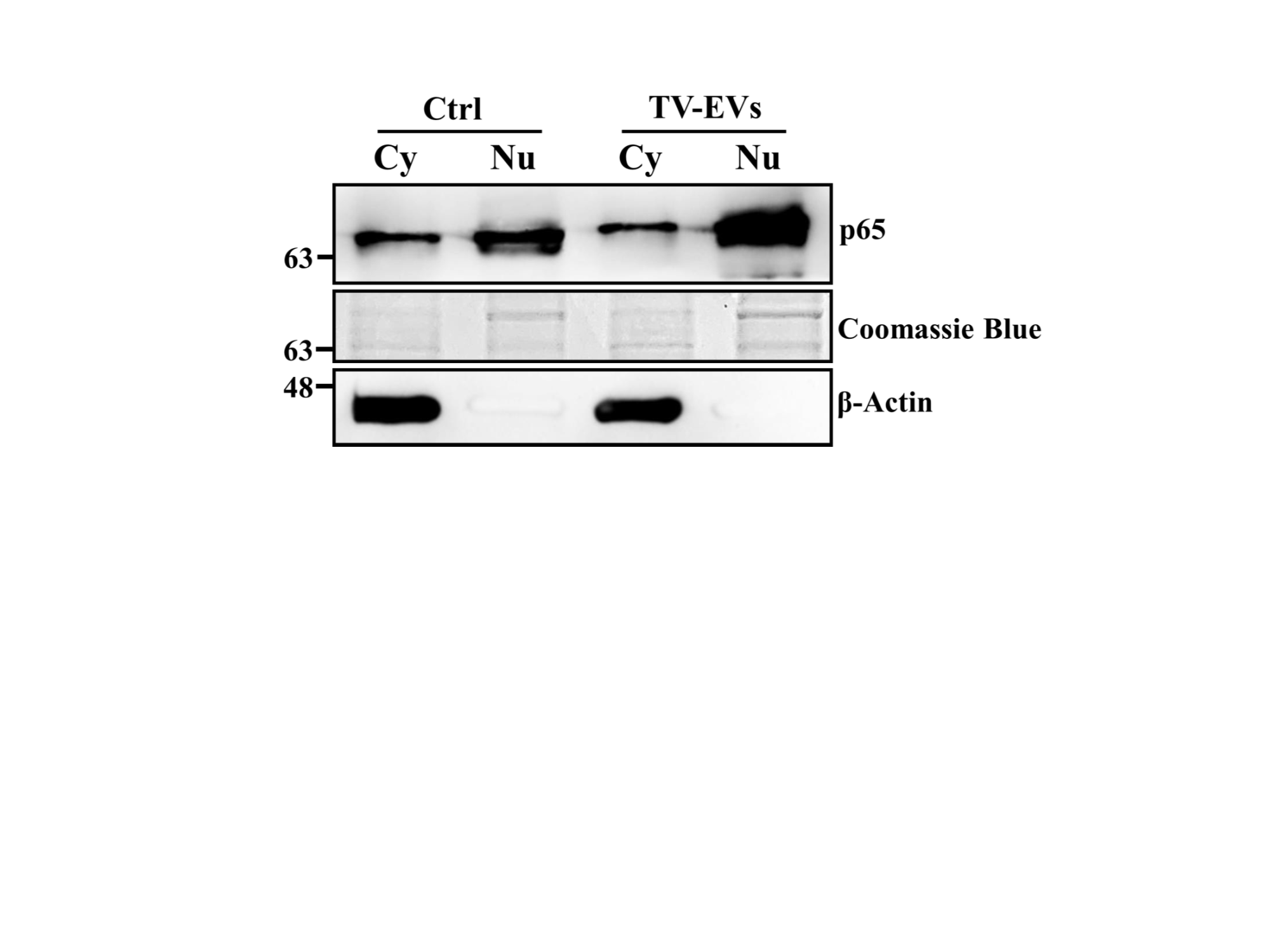

Supplement: S2 Fig — THP-1 macrophages (5 × 10⁶ cells/ml) were treated with the PBS control (Ctrl) or TV-EVs (ATCC 50143) for 4 hours and the expresssion of NF-κB p65 was analysed in the cytoplasmic (Cy) and nuclear (Nu) fractions using western blot. Coomassie blue staining served as the loading control, and β-actin was used as a cytoplasmic marker to confirm fraction purity. (TIFF) [file ppat.1013216.s002.tiff]

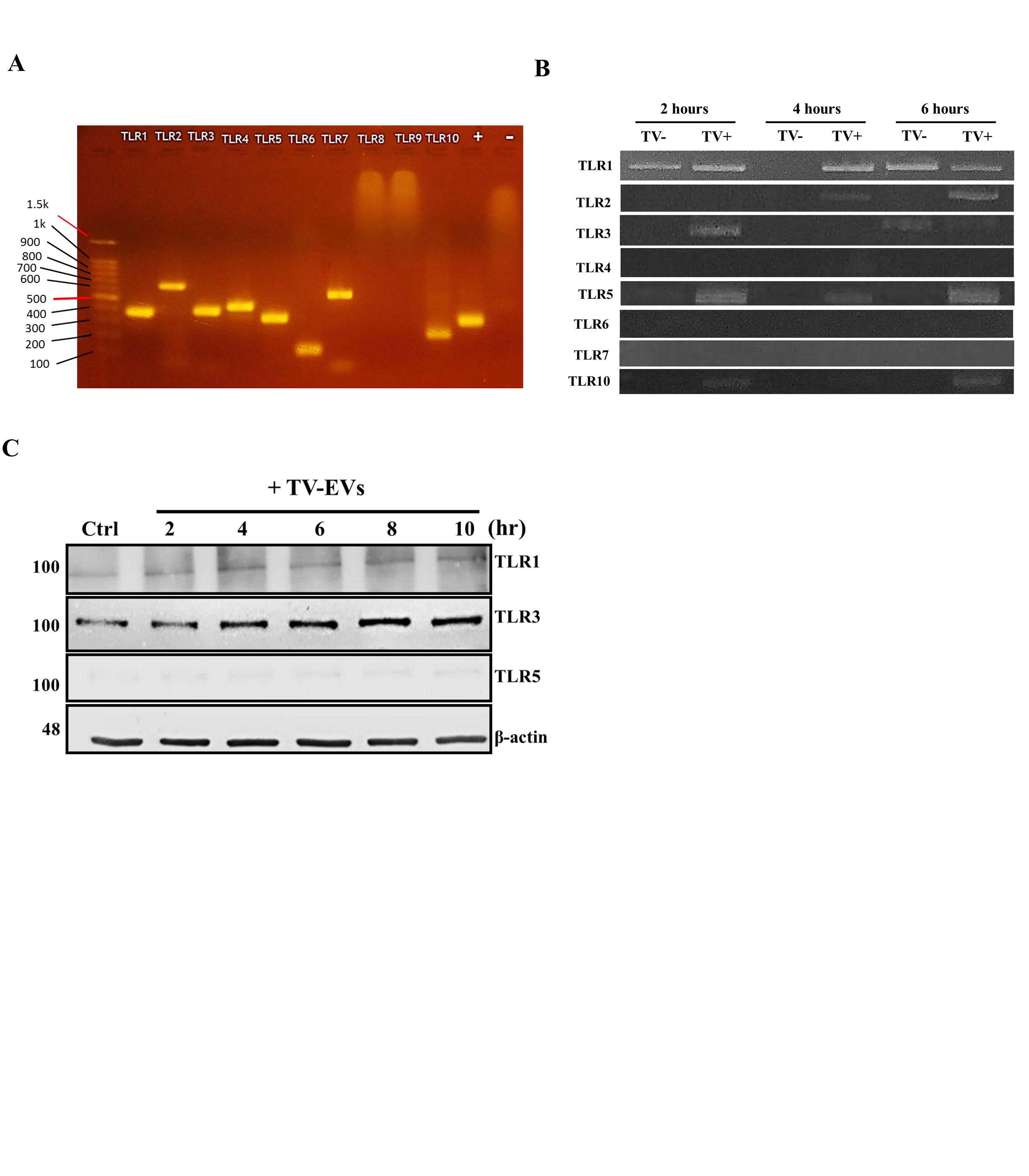

Supplement: S3 Fig — (A) The gene expression of TLR1–10 in Ect was screened by RT-PCR using specific primers. (B) The gene expression of TLRs in Ect stimulated with TV for different time intervals was measured by RT-PCR analysis. (C) The protein expression of TLR1, TLR3 and TLR5 in Ect treated with TV-EVs for different time intervals was detected by western blot. (TIF) [file ppat.1013216.s003.tif]
