# Supplementary material for: Trichomonas vaginalis extracellular vesicles activate the NLRP3 inflammasome and TLR3-mediated inflammatory cascades in host cells
Source: PLoS Pathog. 2025 Jun 2;21(6):e1013216. doi: 10.1371/journal.ppat.1013216 (PMC12157993; doi:10.1371/journal.ppat.1013216)
Supplement: S1 Data — This file includes raw image data of Figs 1A, 1B, 4A, 5A, 5D, 6A, 6D, 7A, 7B, 8A, 8B, 9C, 9D and 9E. (PDF) [file ppat.1013216.s007.pdf]

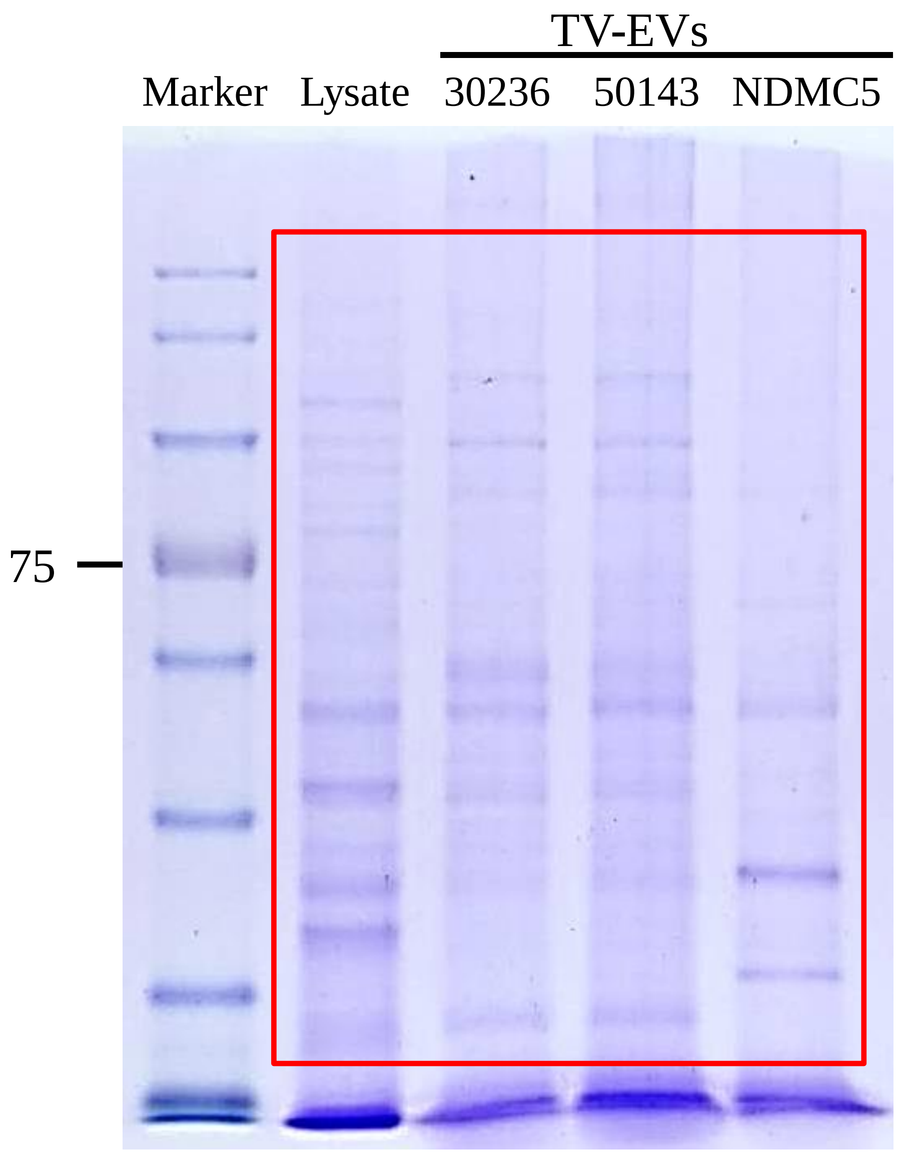

Fig 1A. The raw data of SDS PAGE with Coomassie Blue staining
The boxed regions were shown in the article.

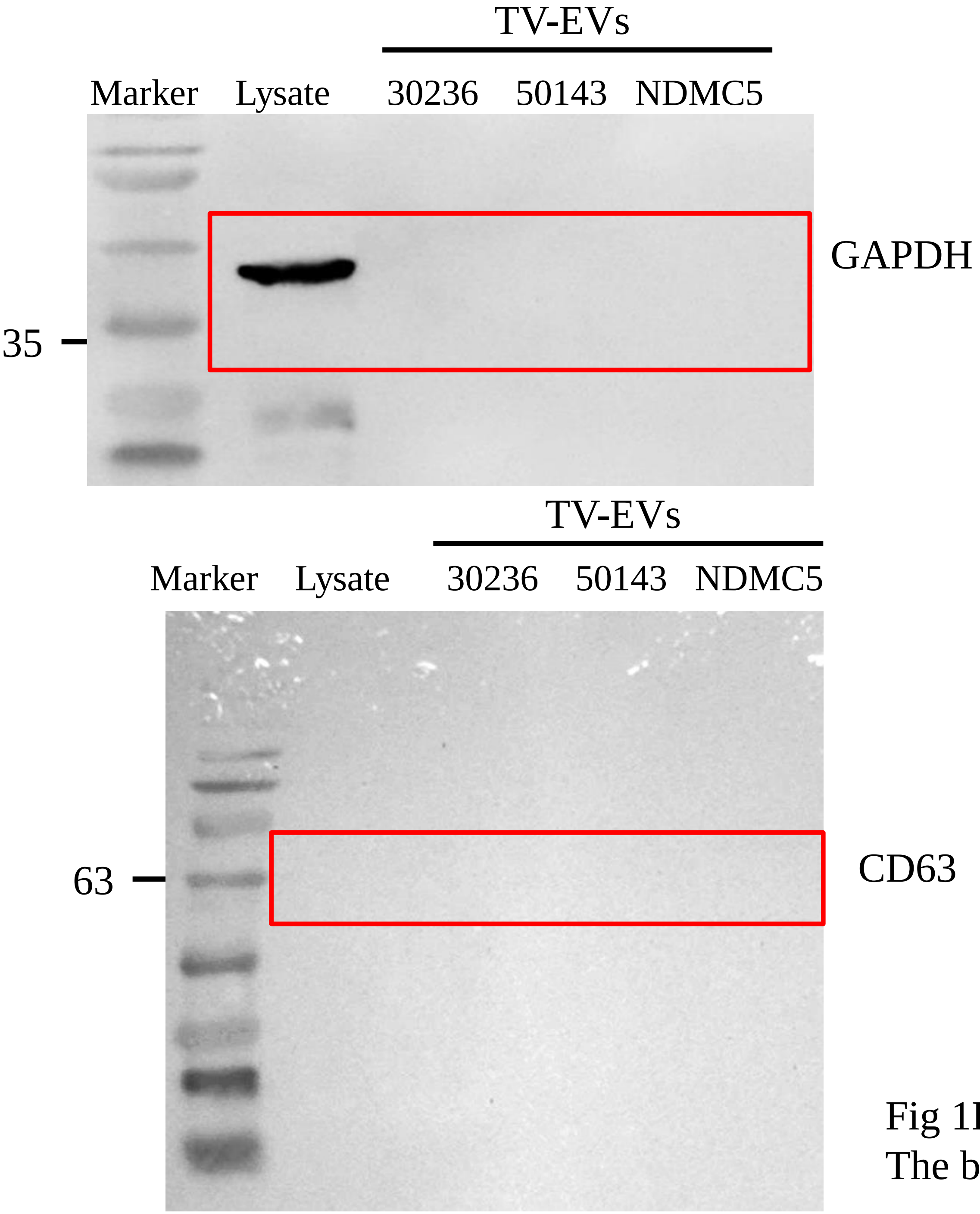

Fig 1B. The raw data of western blotting.
The boxed regions were shown in the article.

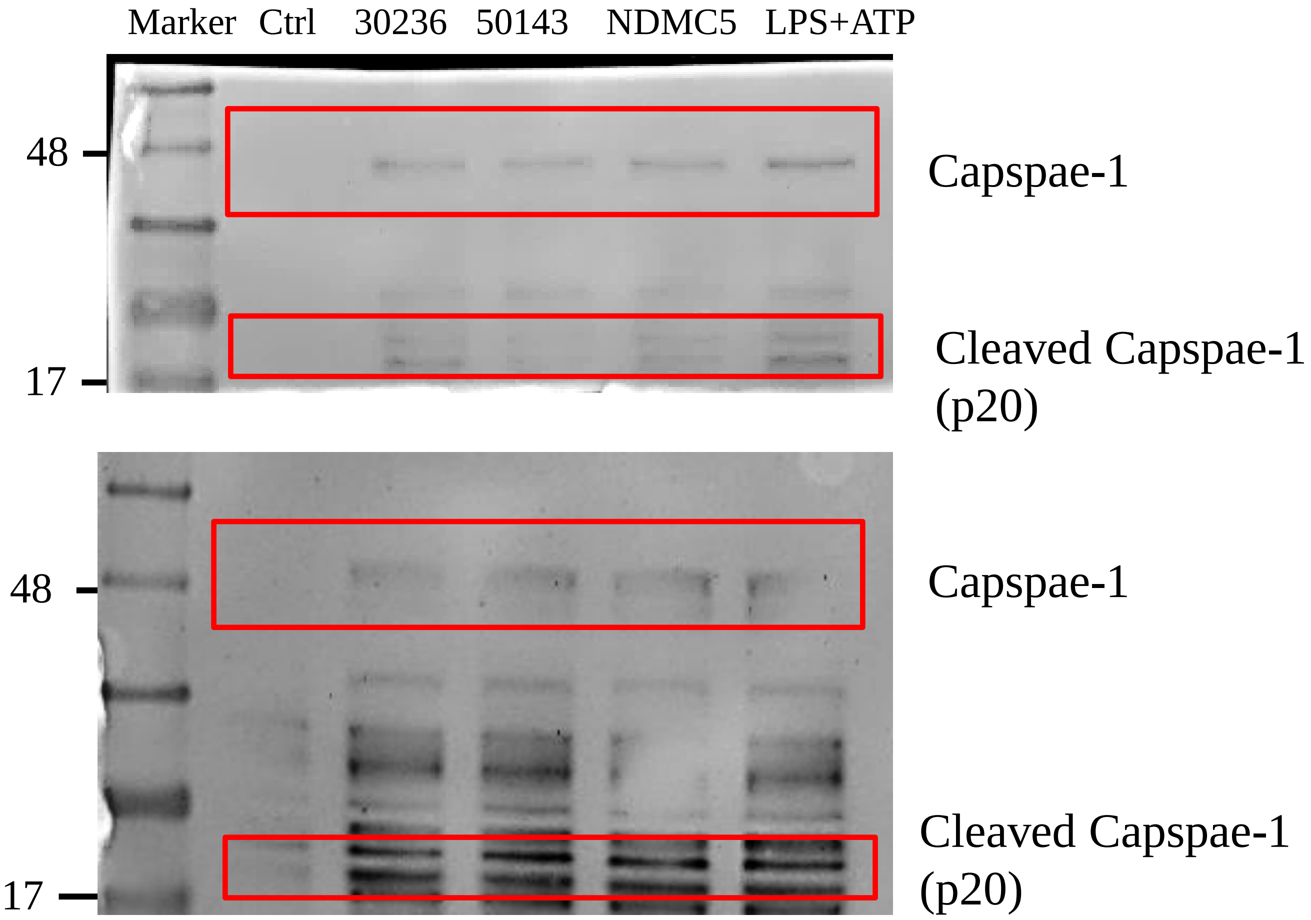

Fig 4A. The raw data of western blotting.
The boxed regions were shown in the article.

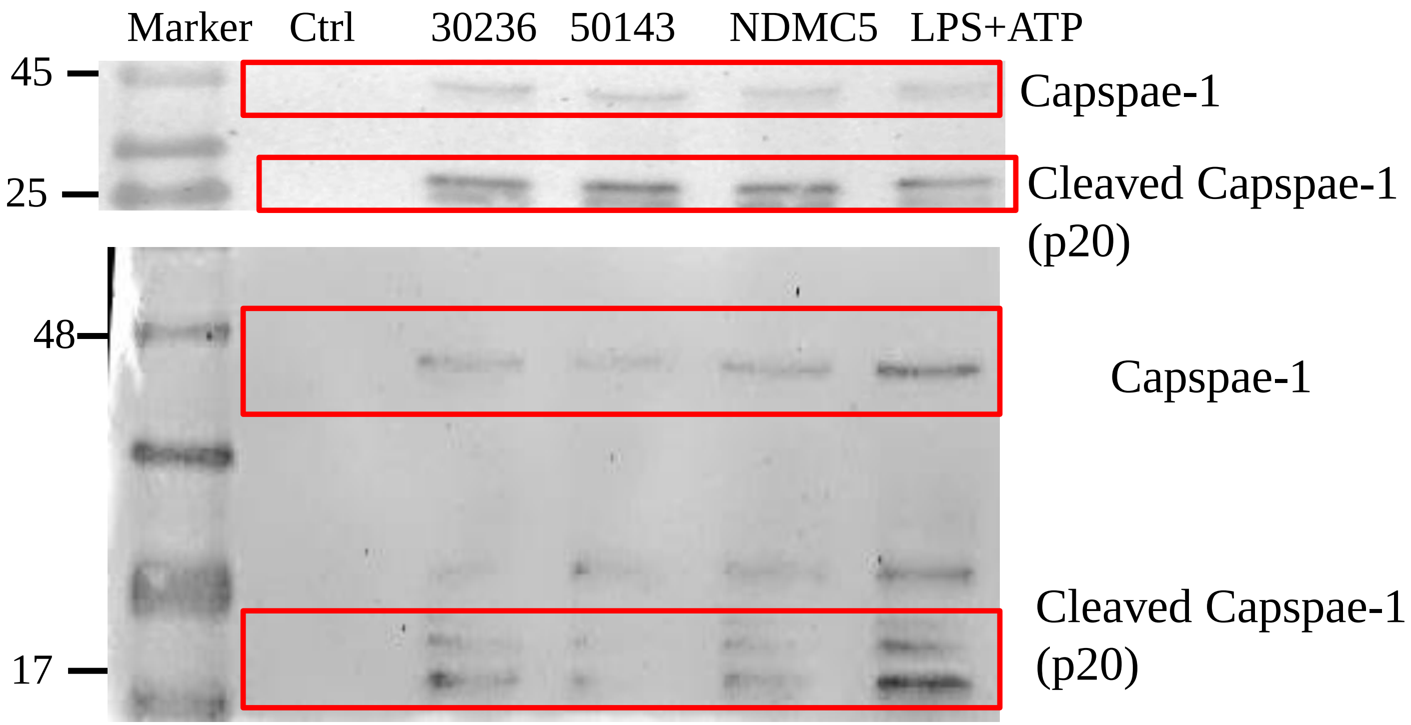

Fig 4A. The raw data of western blotting.
The boxed regions were shown in the article.

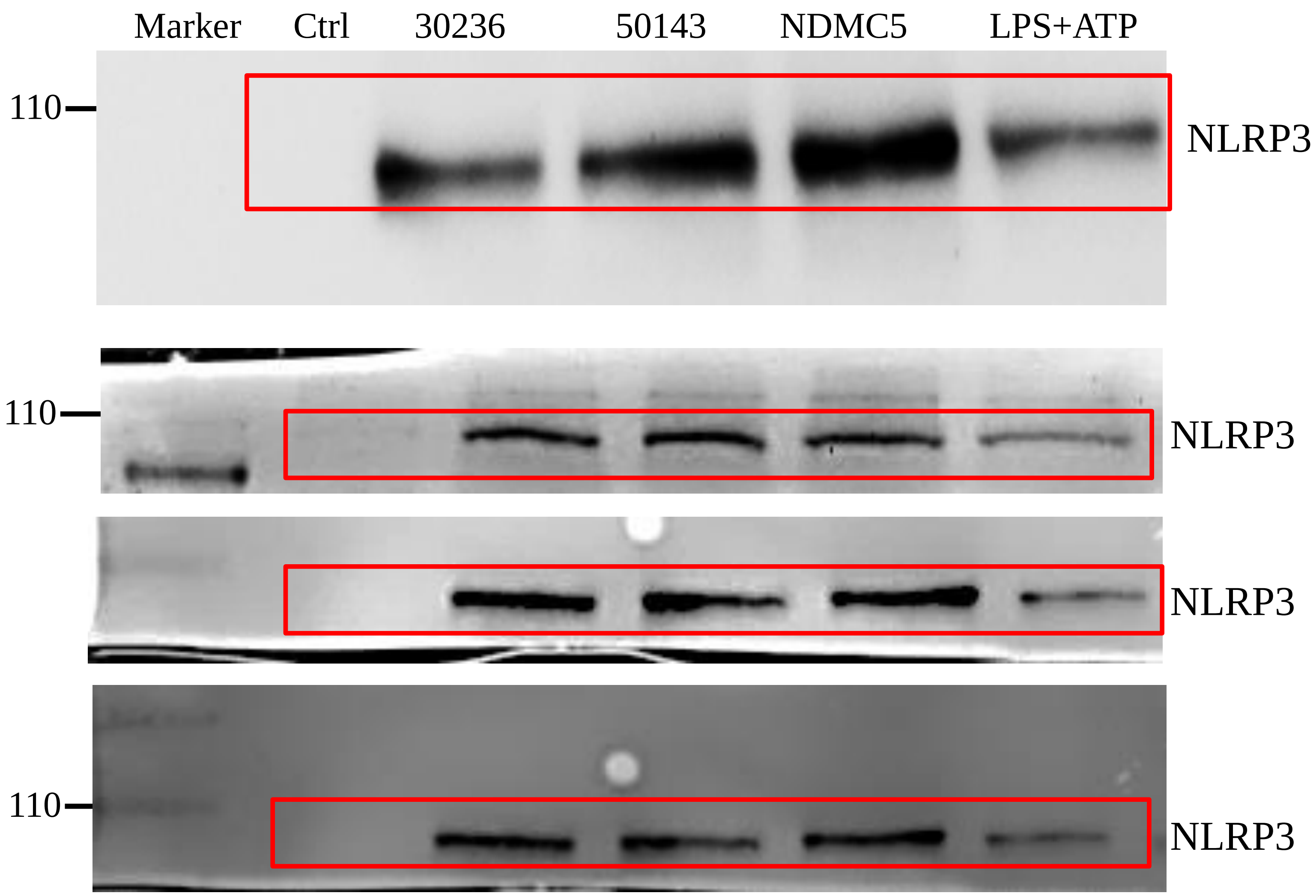

Fig 4A. The raw data of western blotting.
The boxed regions were shown in the article.

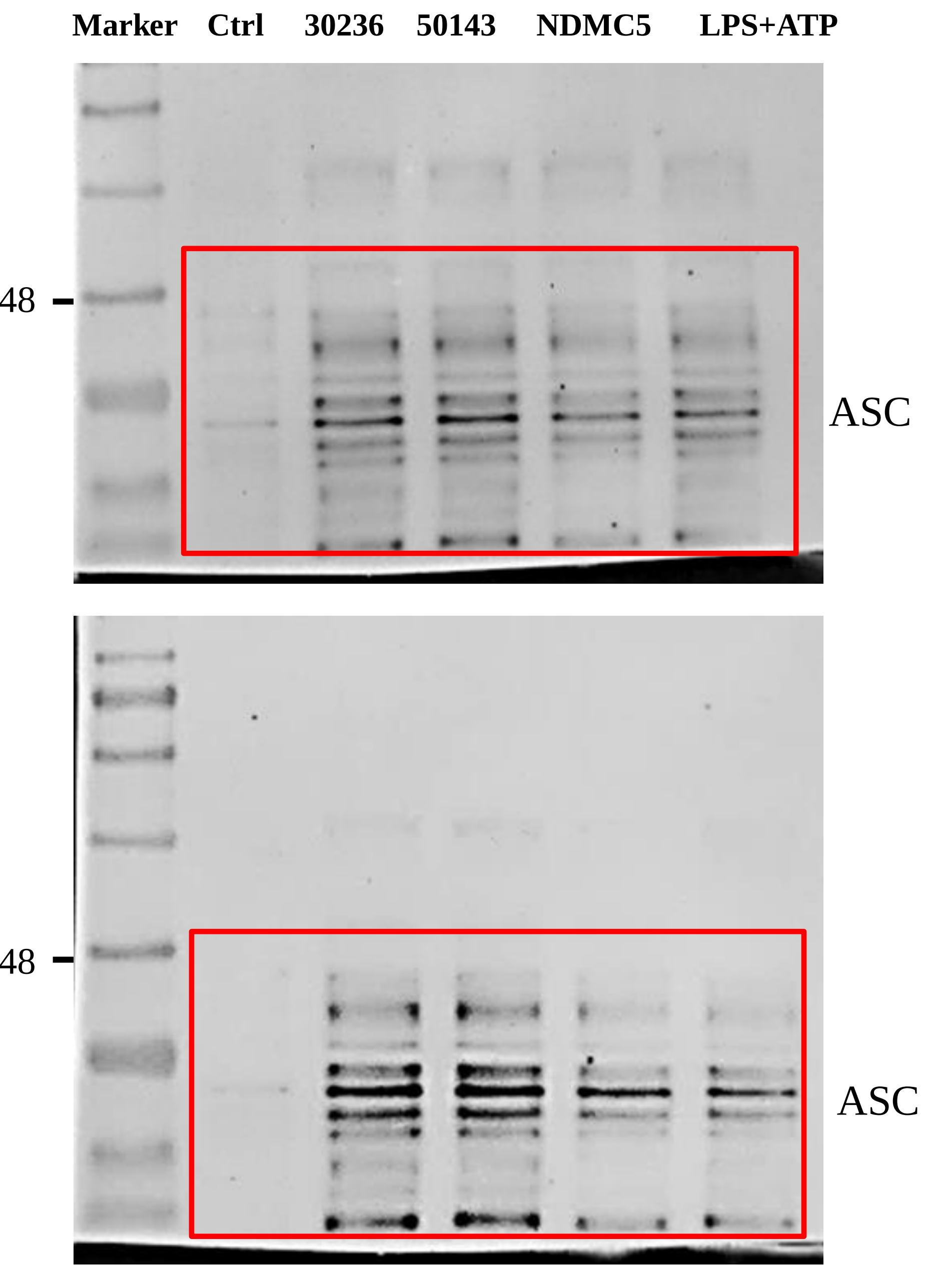

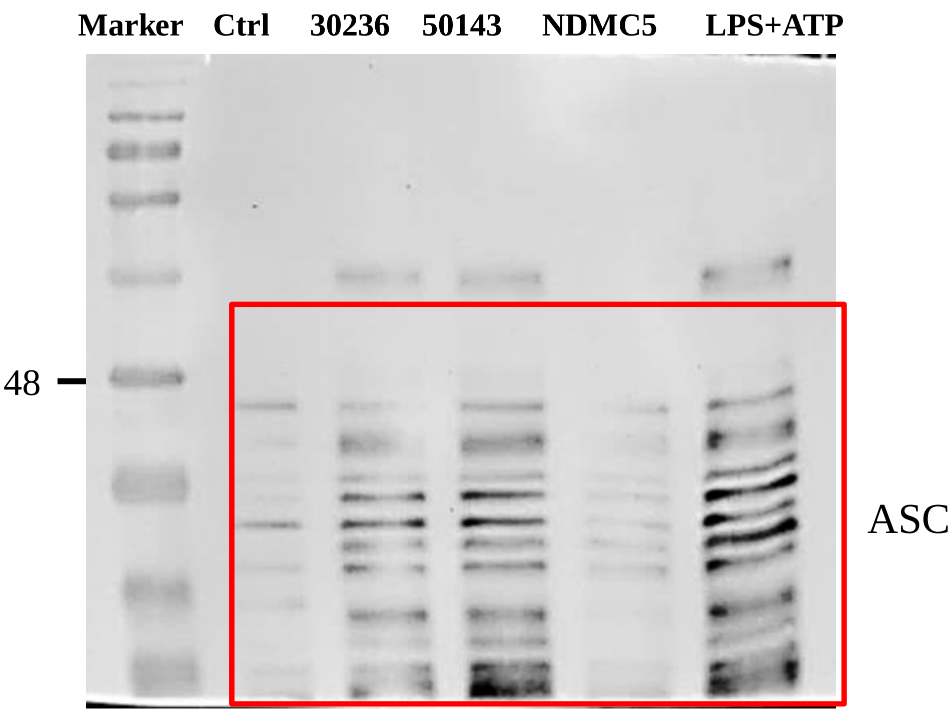

Fig 4A. The raw data of western blotting. The boxed regions were shown in the article.

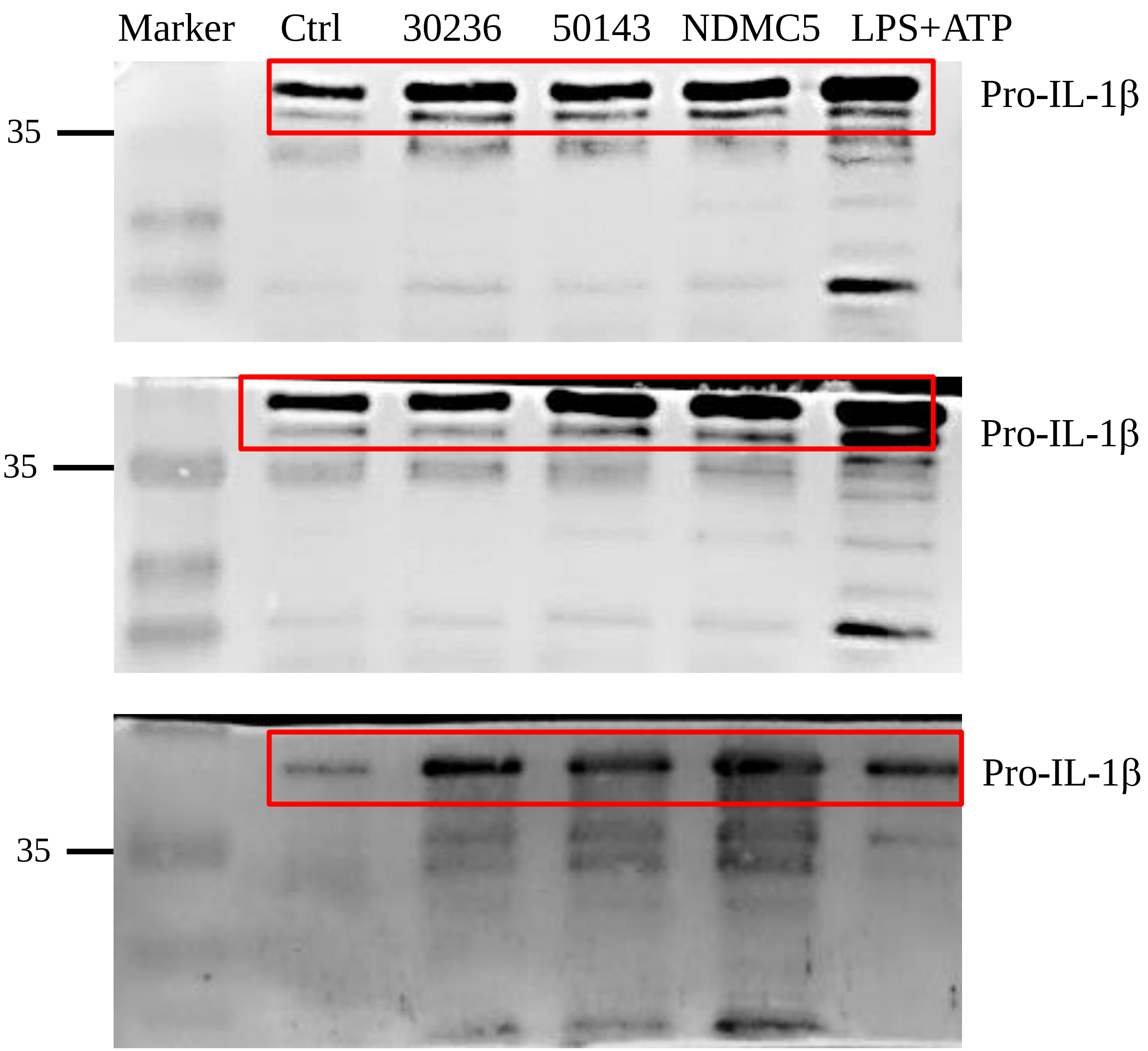

Fig 4A. The raw data of western blotting.
The boxed regions were shown in the article.

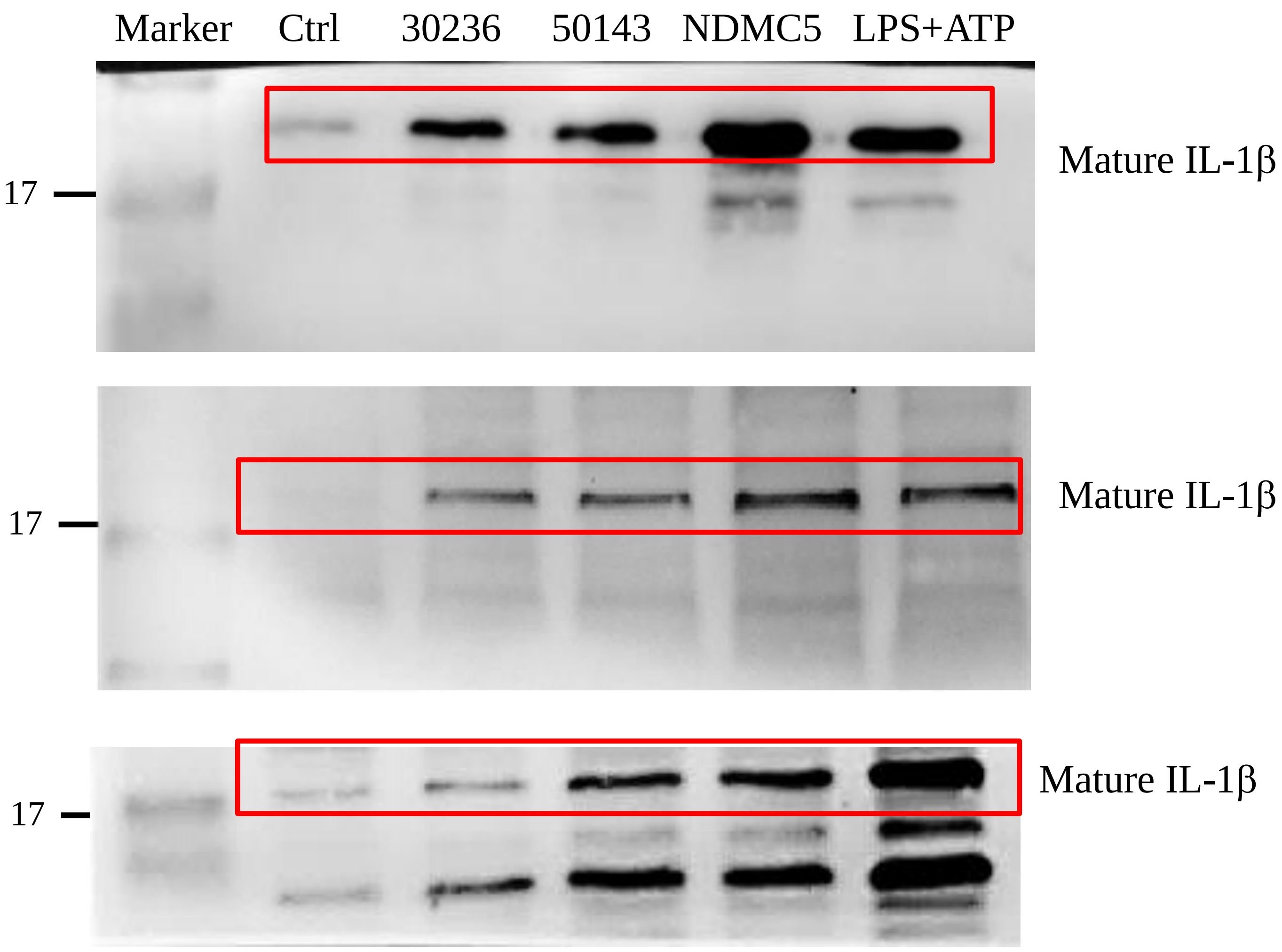

Fig 4A. The raw data of western blotting.
The boxed regions were shown in the article.

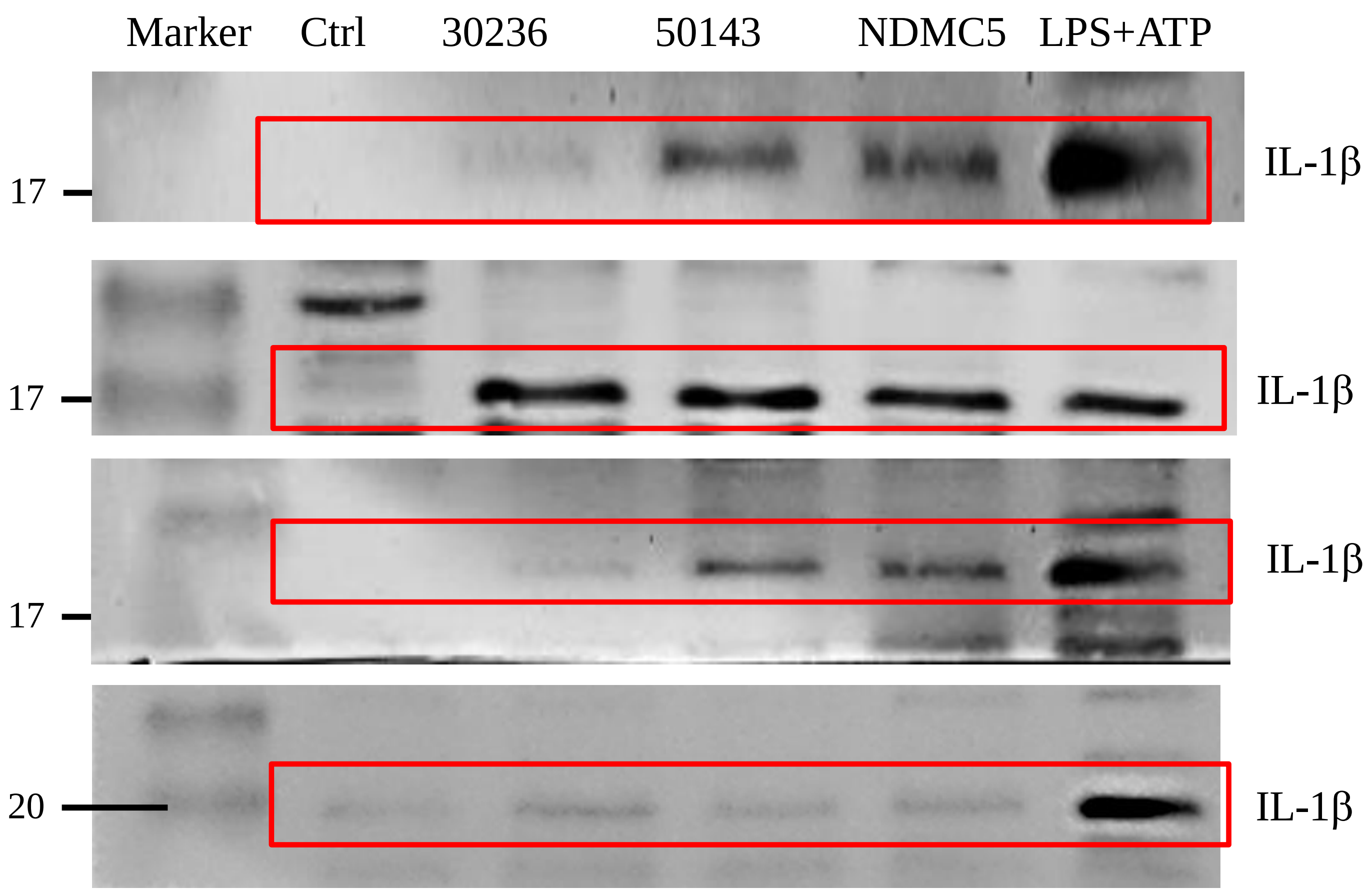

Fig 4A. The raw data of western blotting.
The boxed regions were shown in the article

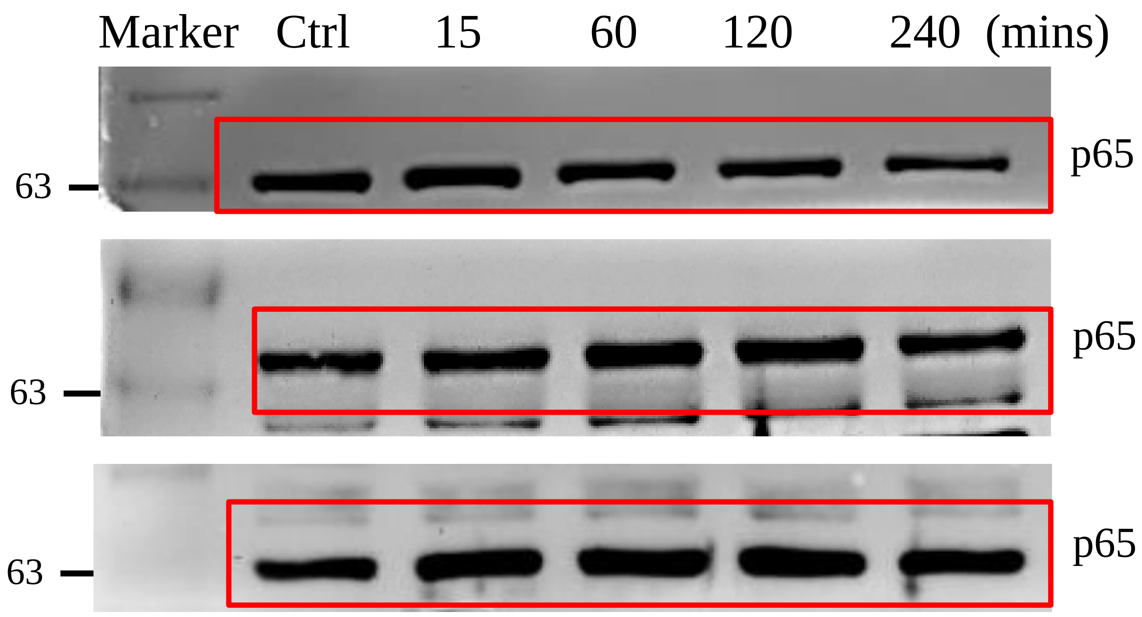

Fig 5A. The raw data of western blotting.
The boxed regions were shown in the article

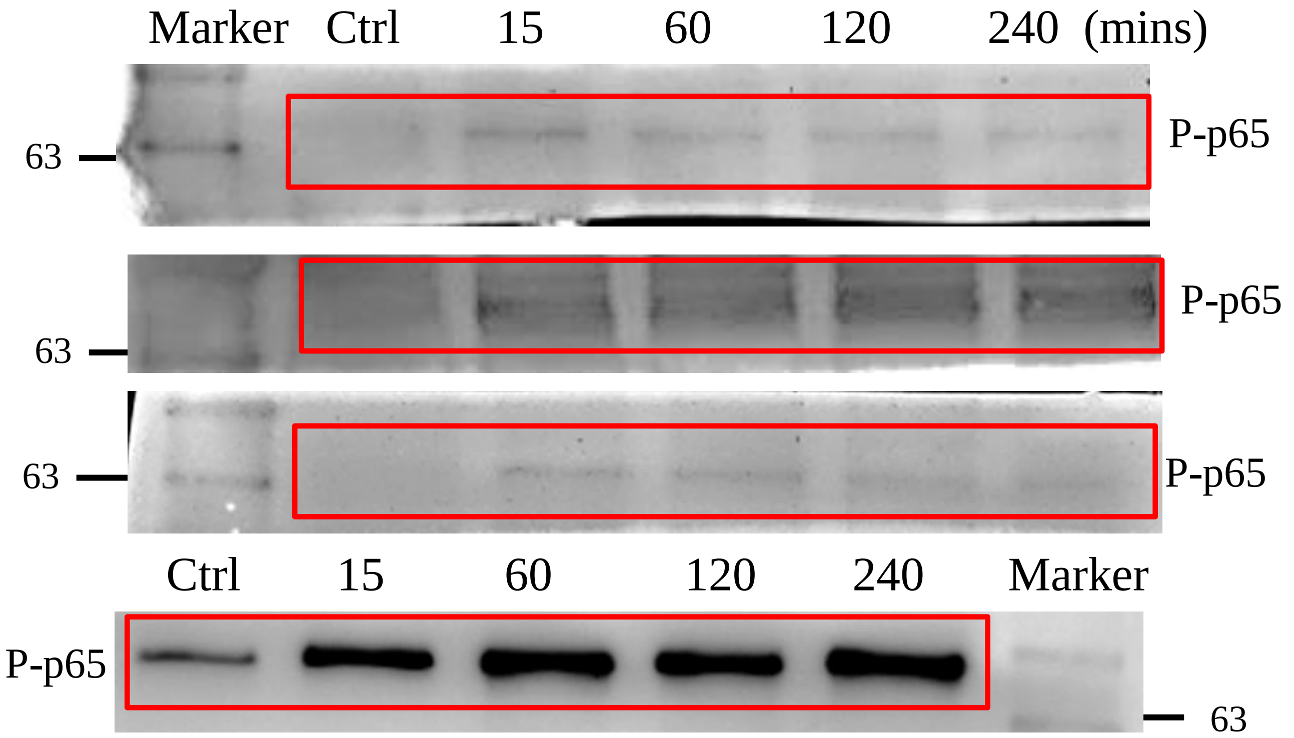

Fig 5A. The raw data of western blotting.
The boxed regions were shown in the article

Fig 5D. The raw data of western blotting. The boxed regions were shown in the article.

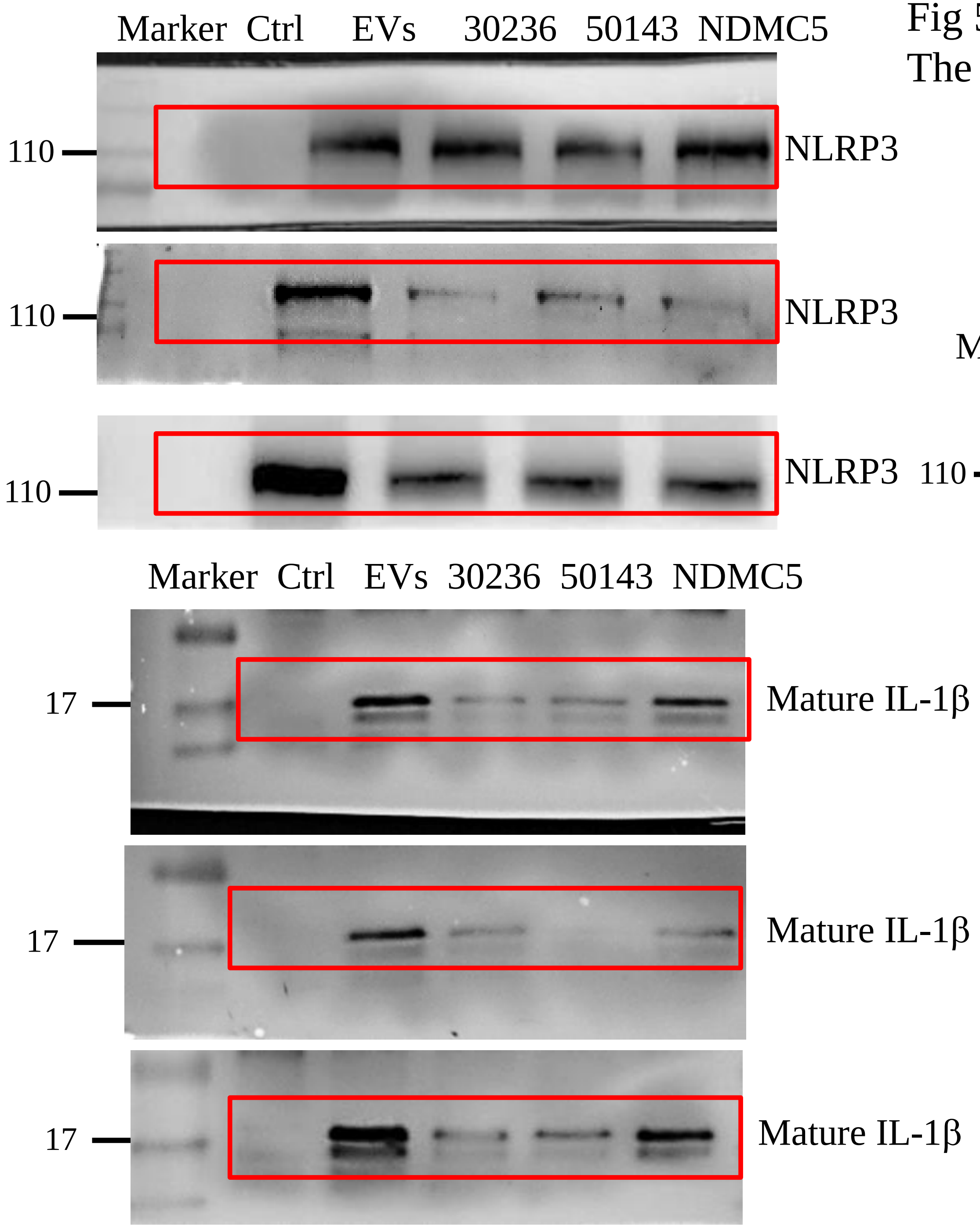

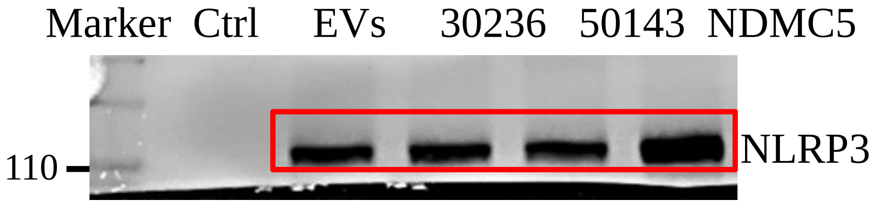

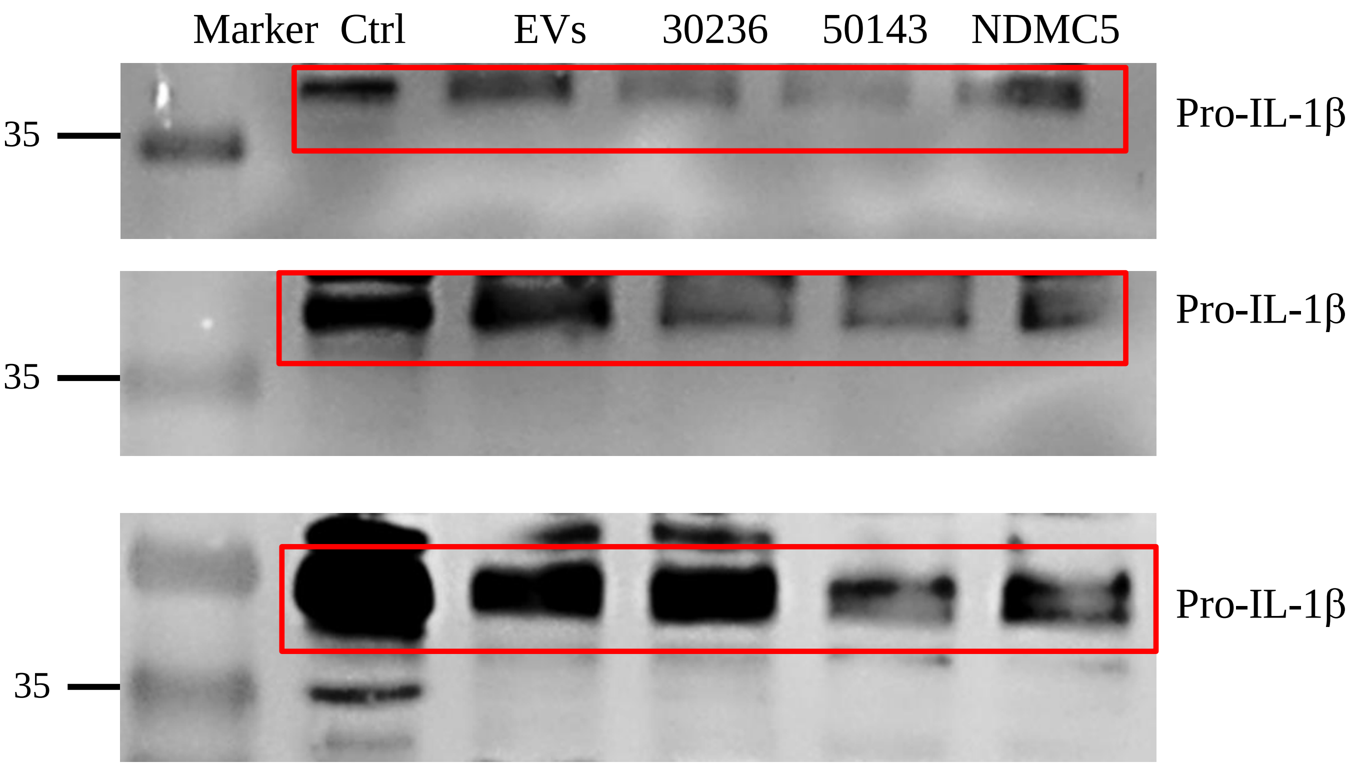

Fig 5D. The raw data of western blotting.
The boxed regions were shown in the article.

Fig 6A. The raw data of western blotting.
The boxed regions were shown in the article.

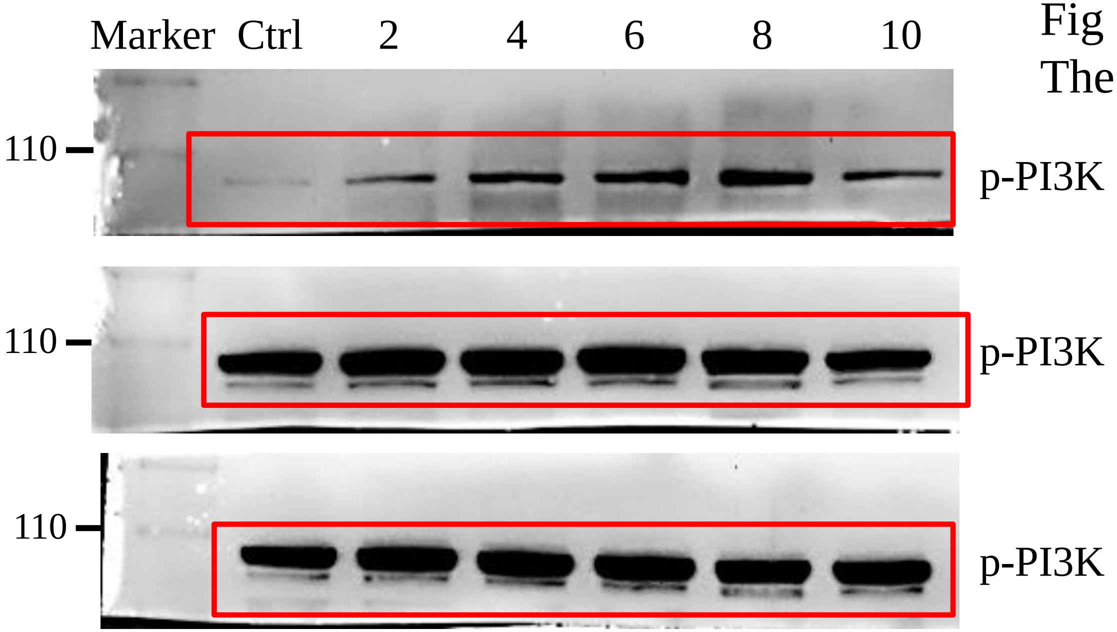

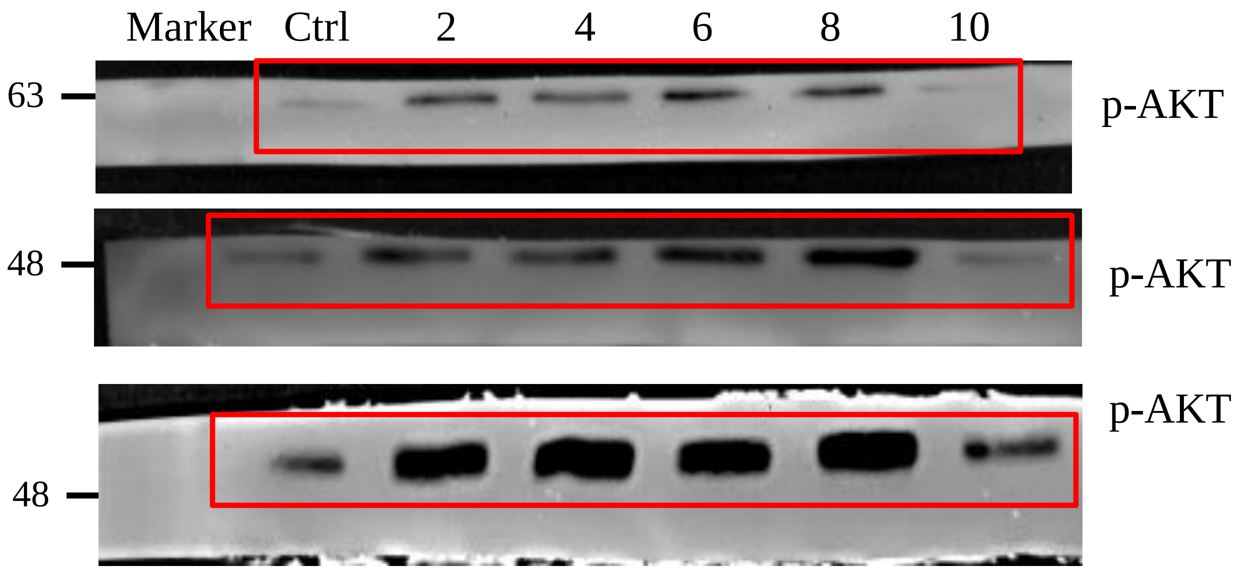

Fig 6A. The raw data of western blotting. The boxed regions were shown in the article.

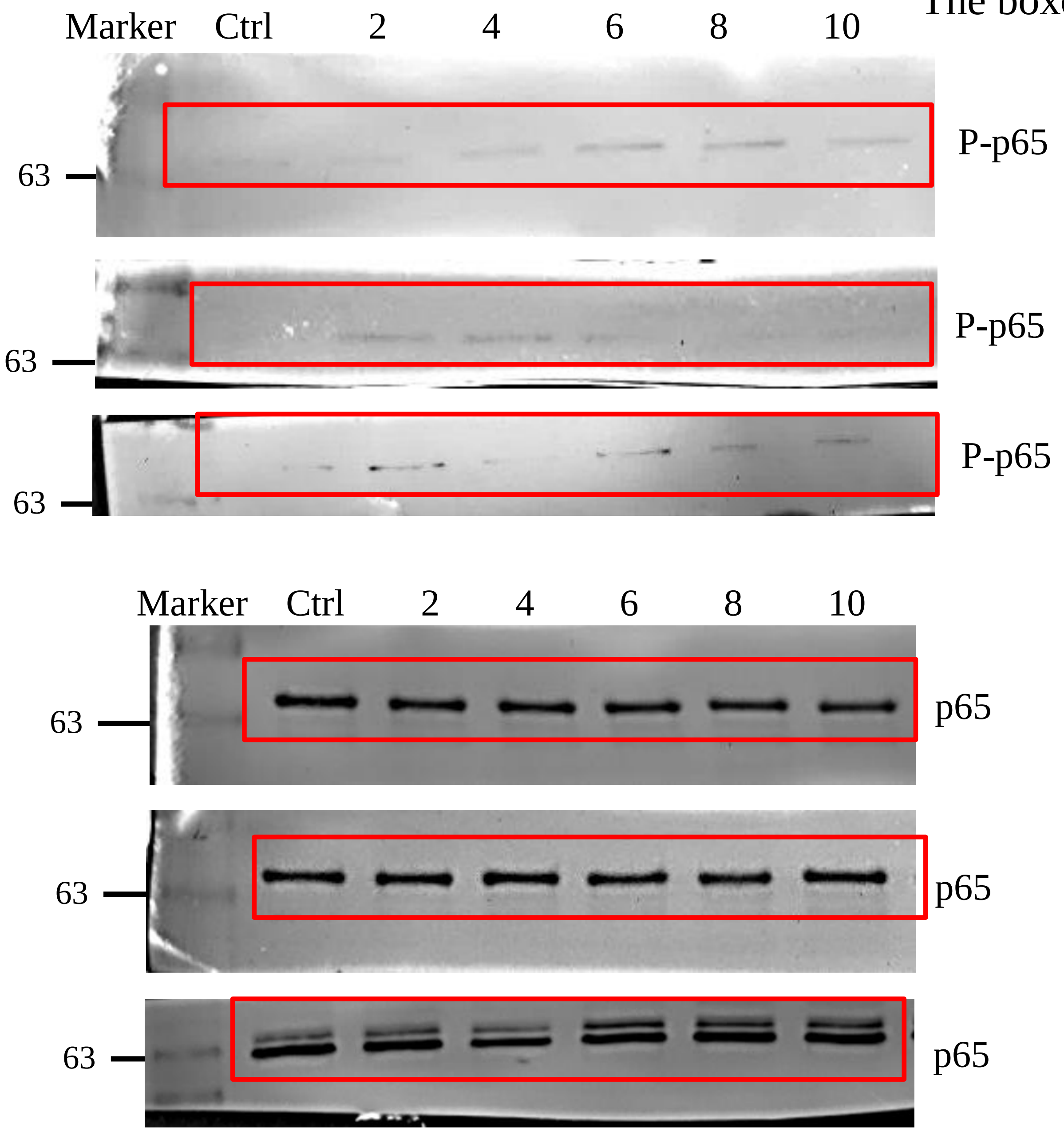

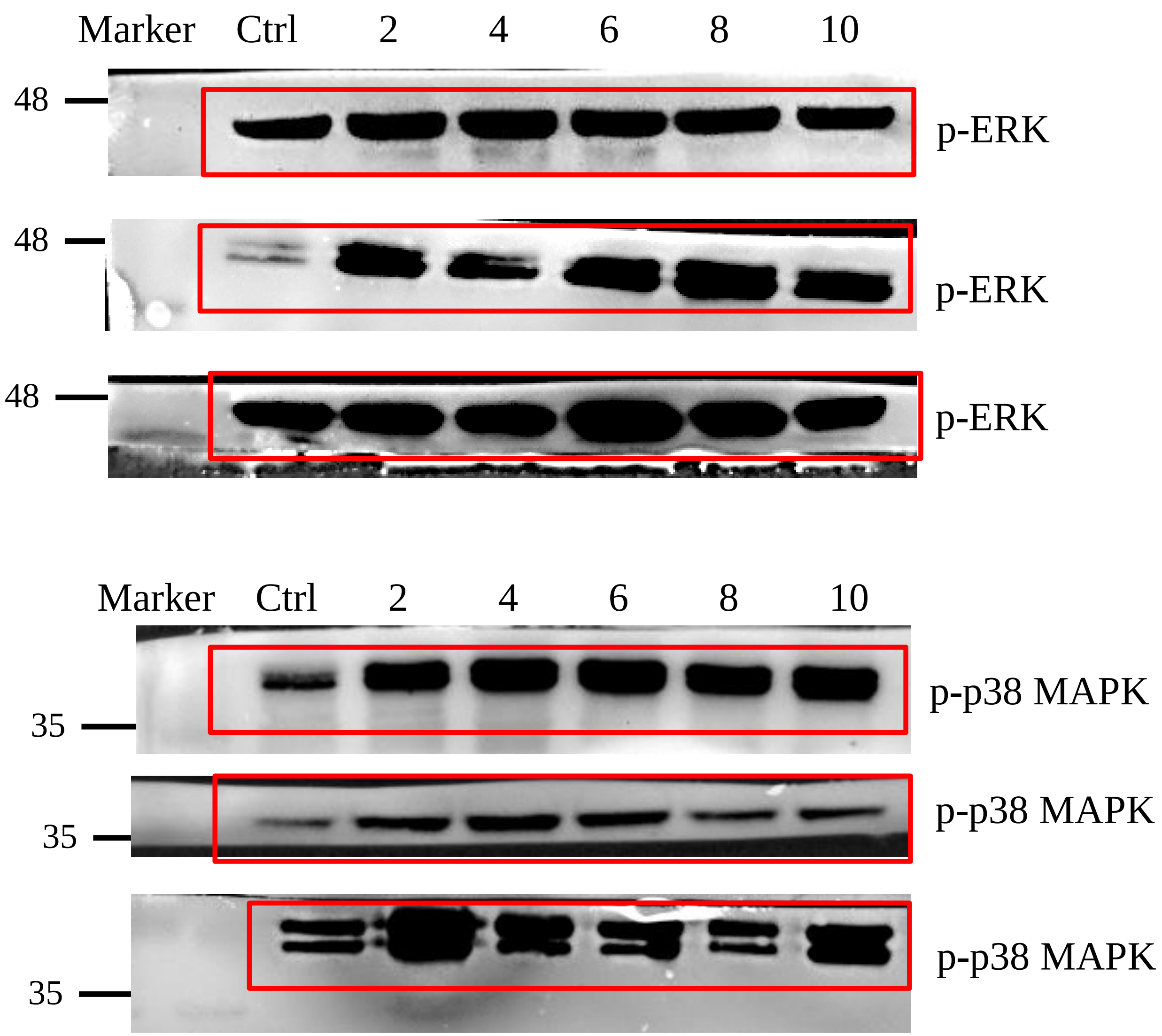

Fig 6A. The raw data of western blotting.
The boxed regions were shown in the article.

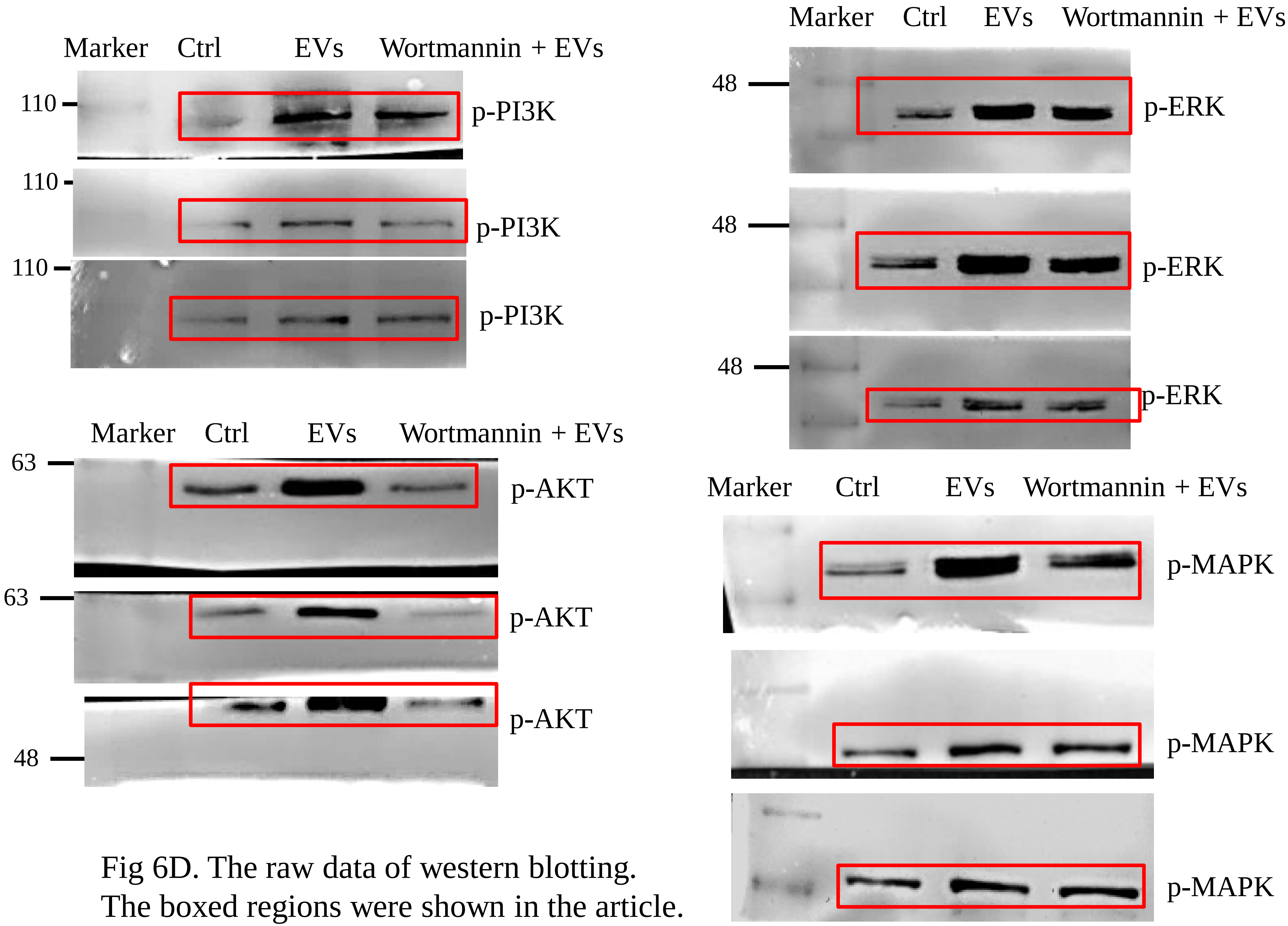

Fig 6D. The raw data of western blotting.
The boxed regions were shown in the article.

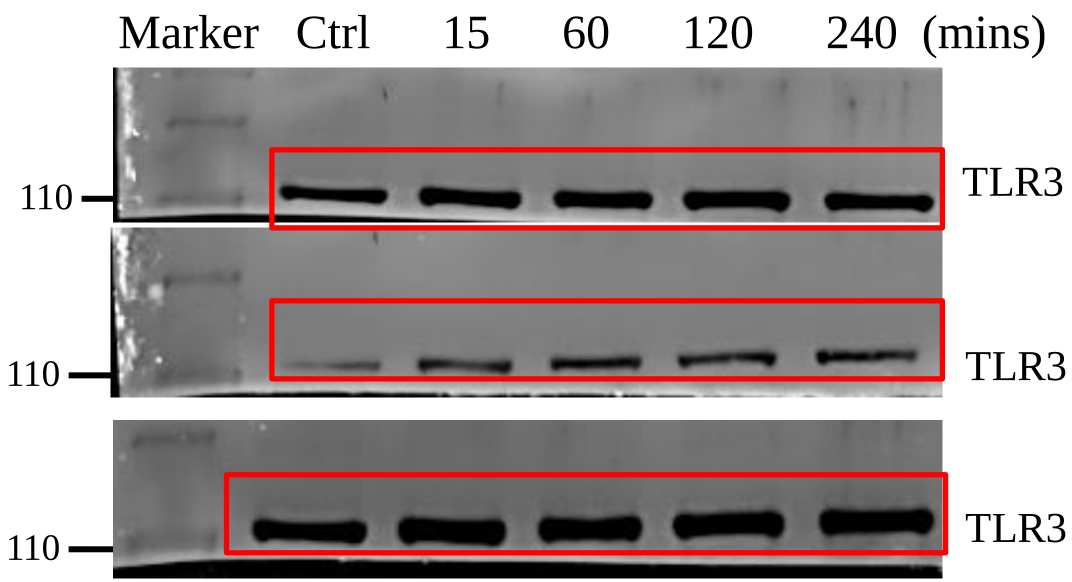

Fig 7A. The raw data of western blotting.
The boxed regions were shown in the article.

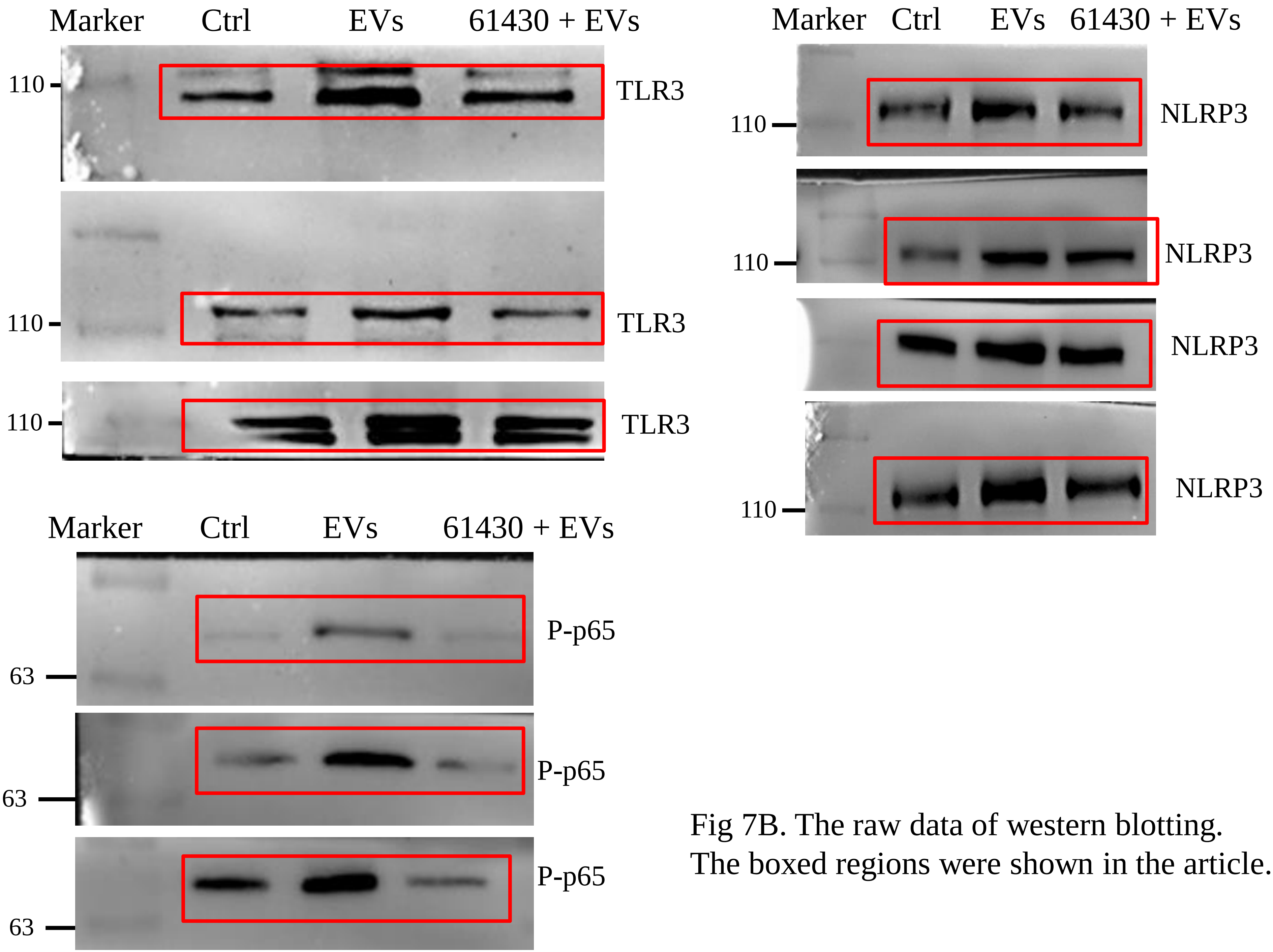

Fig 7B. The raw data of western blotting.
The boxed regions were shown in the article.

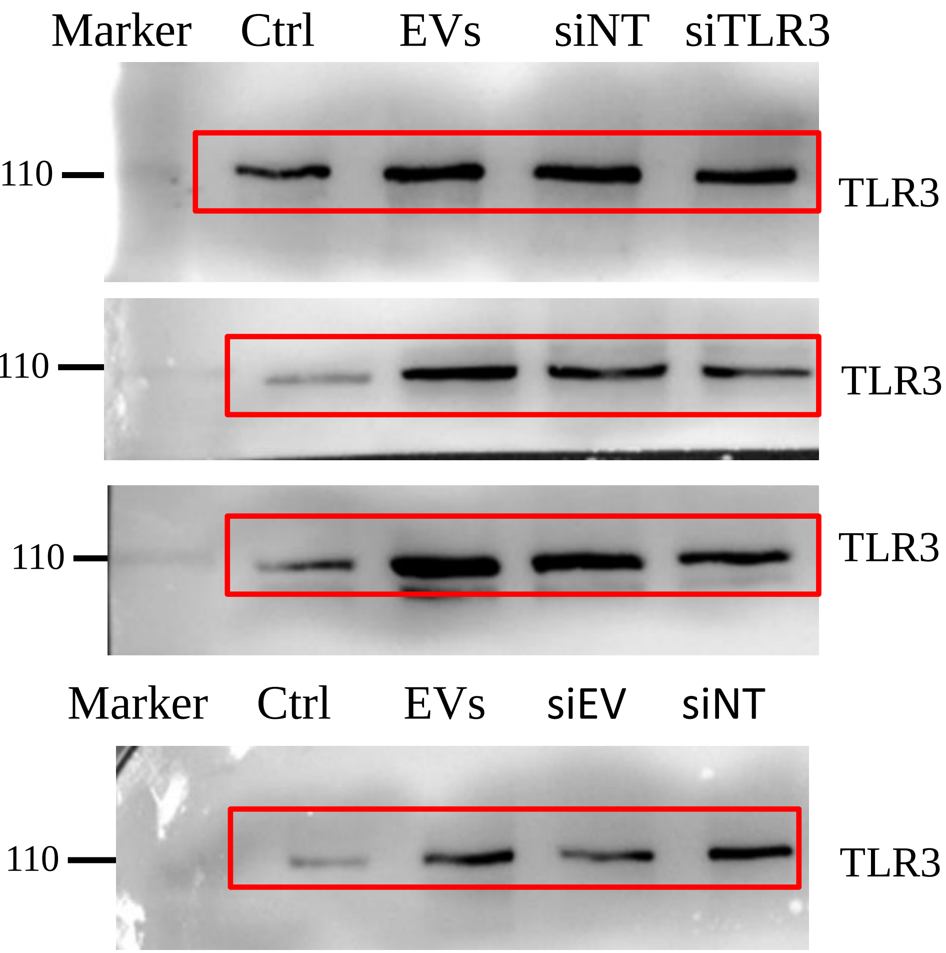

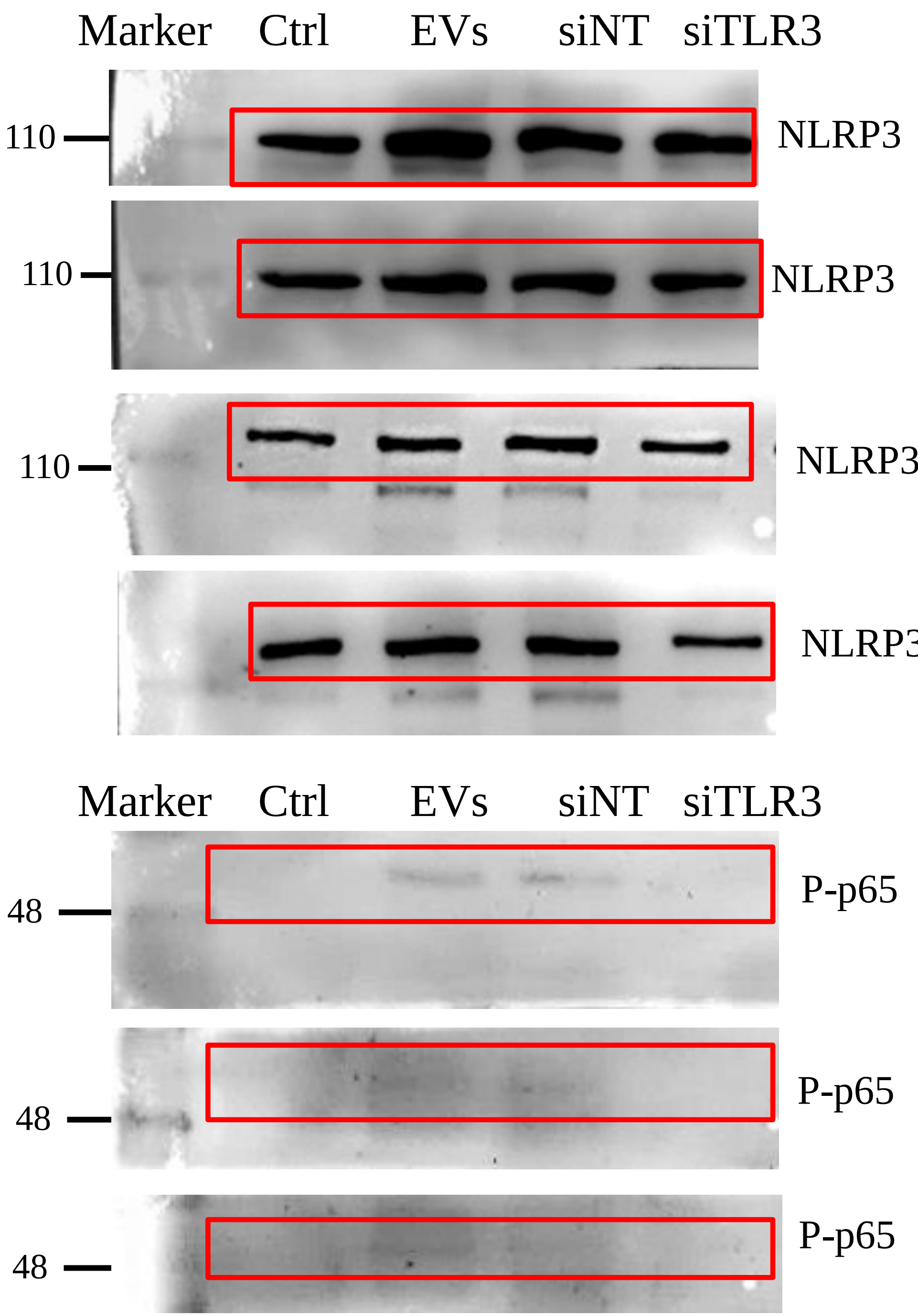

Fig 7B. The raw data of western blotting.
The boxed regions were shown in the article.

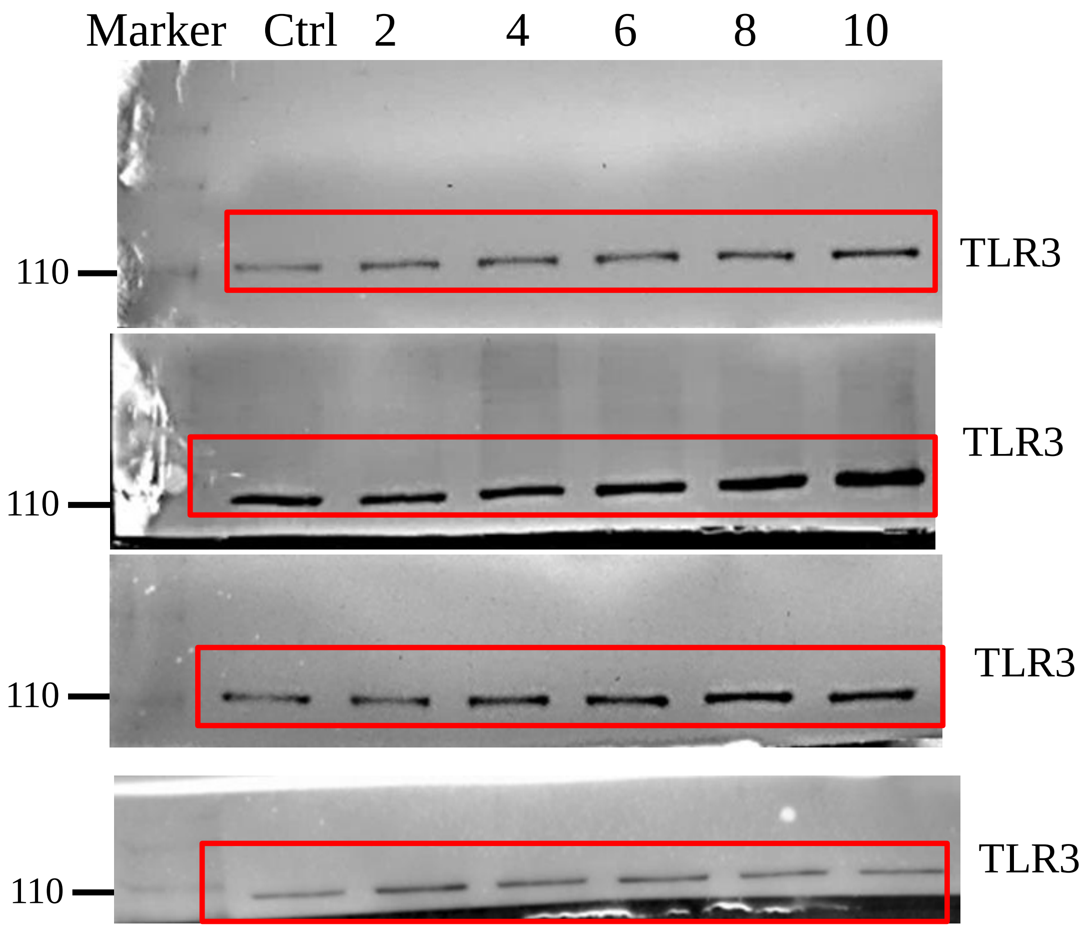

Fig 8A. The raw data of western blotting.
The boxed regions were shown in the article.

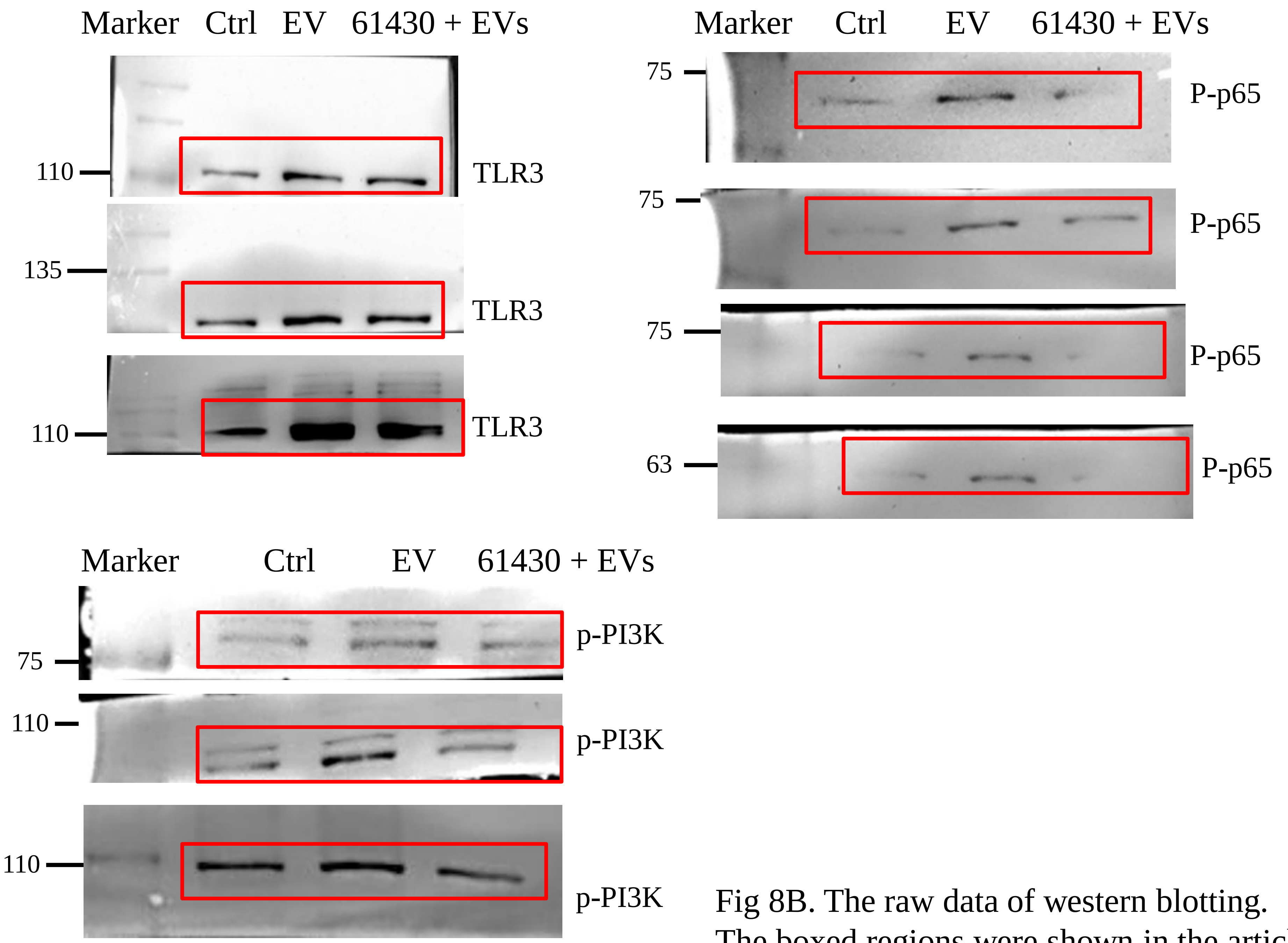

Fig 8B. The raw data of western blotting.
The boxed regions were shown in the article.

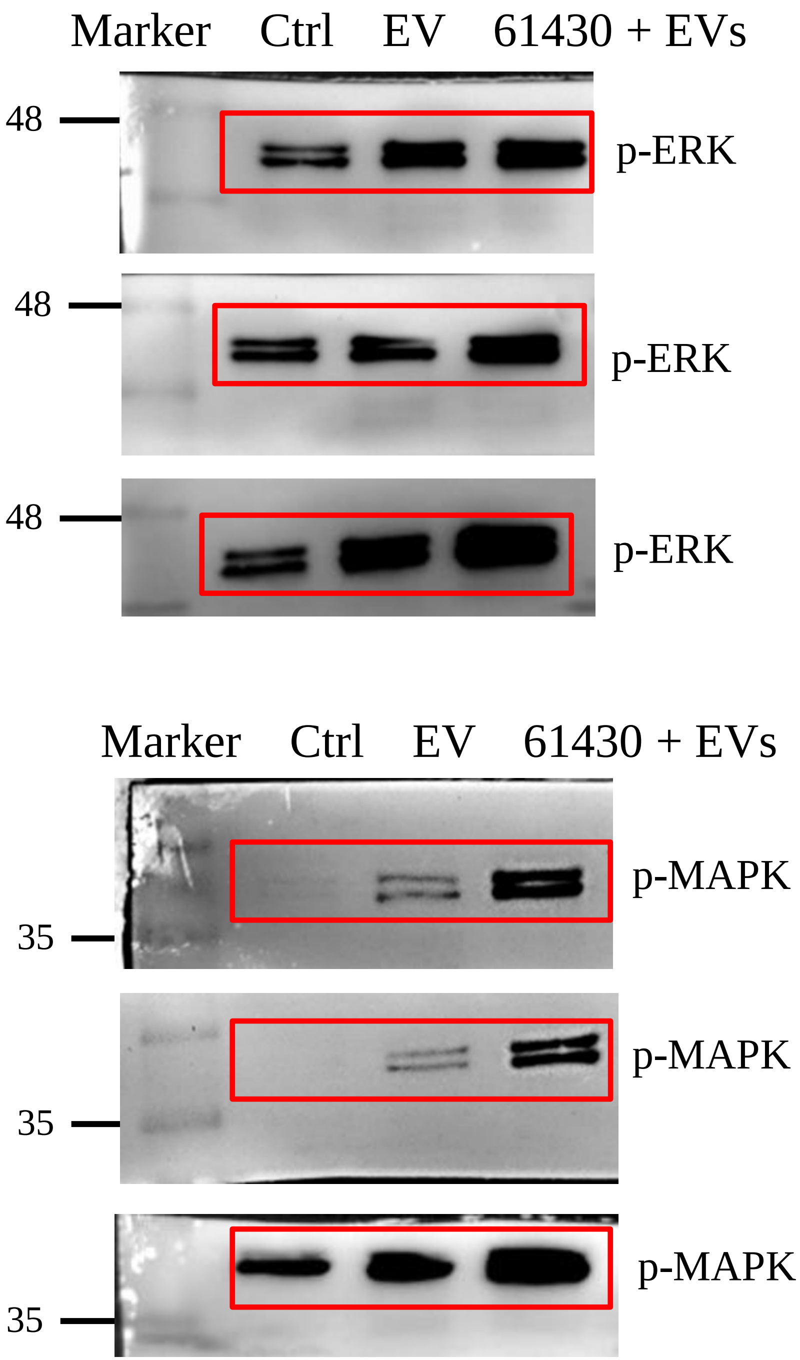

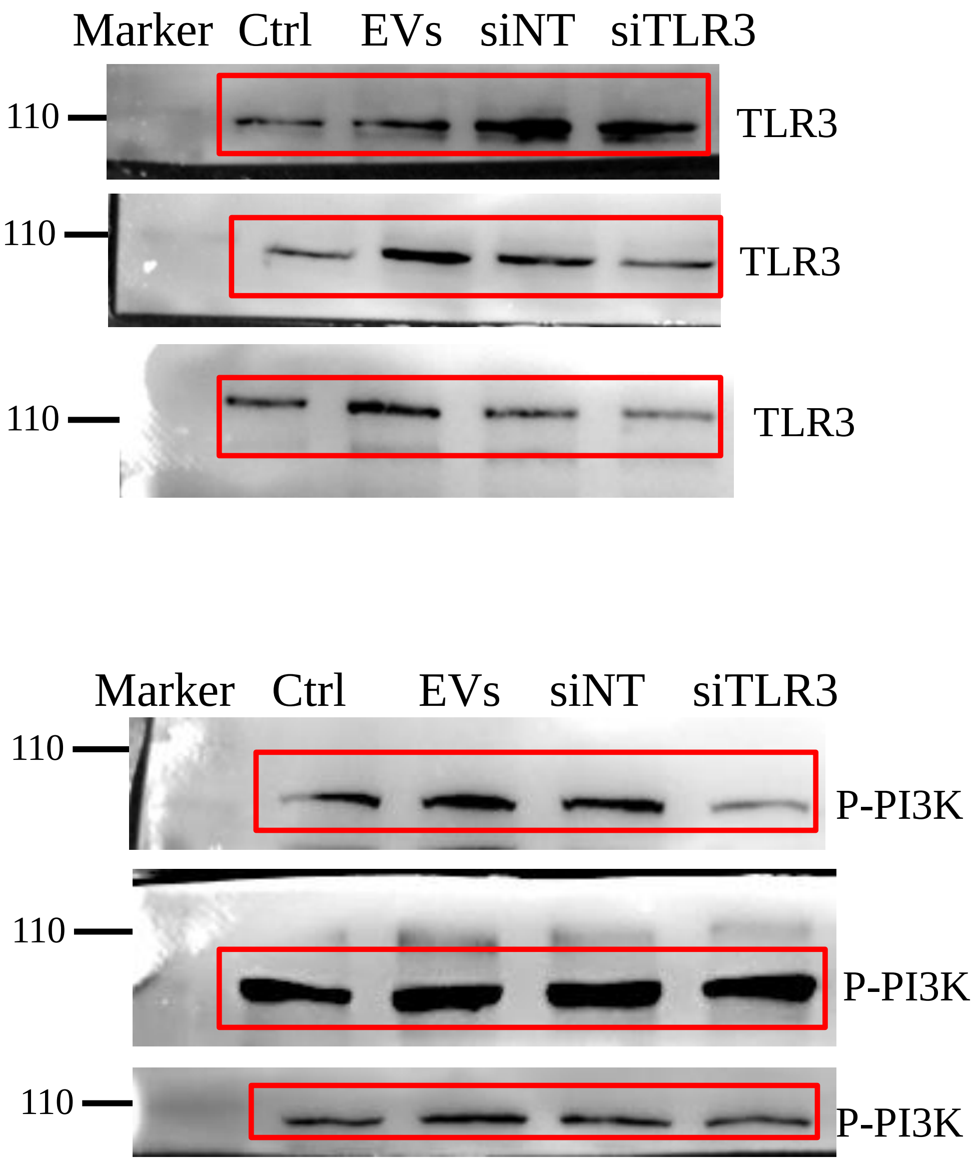

Fig 8B. The raw data of western blotting.
The boxed regions were shown in the article.

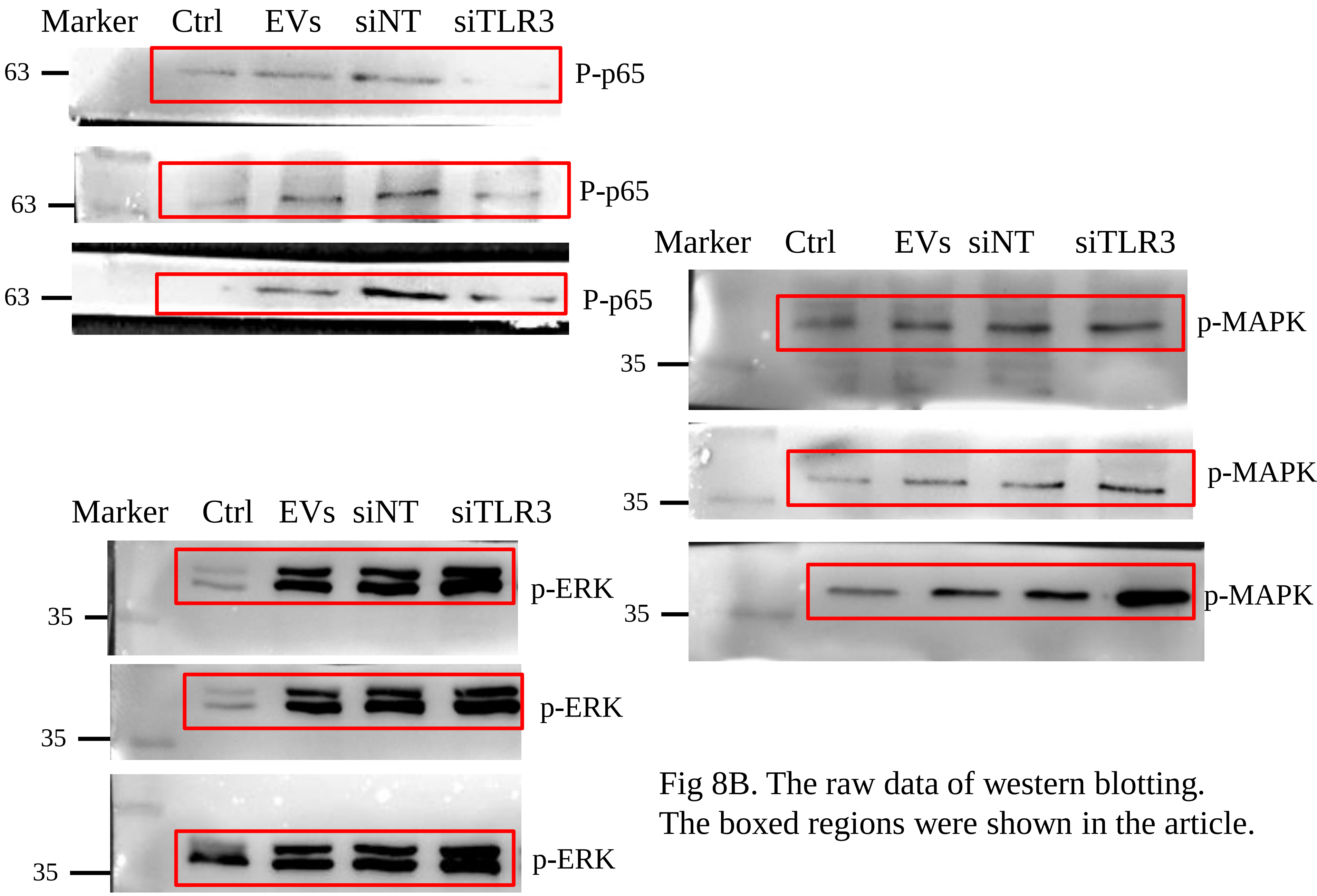

Fig 8B. The raw data of western blotting.
The boxed regions were shown in the article.

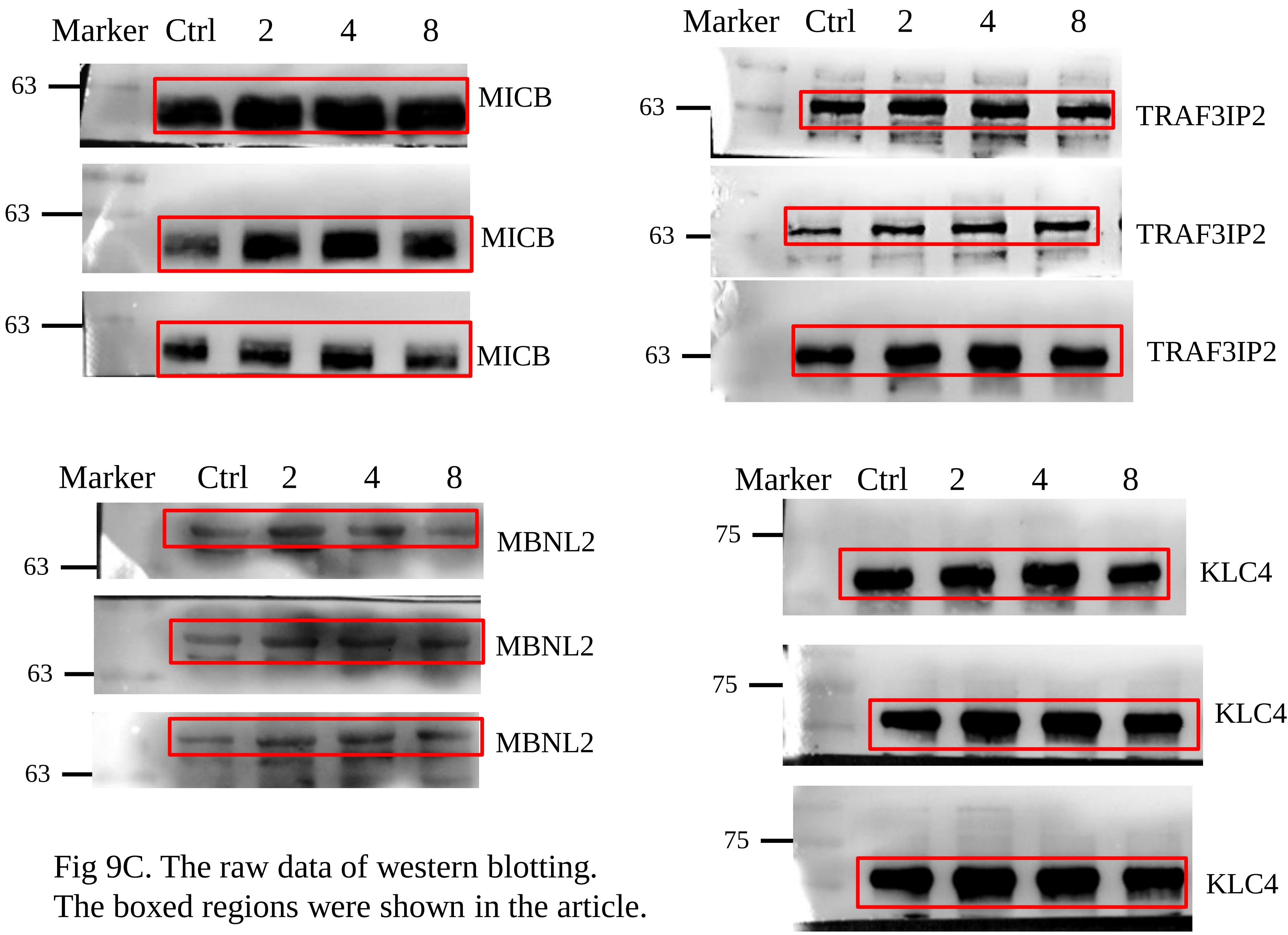

Fig 9C. The raw data of western blotting.
The boxed regions were shown in the article.

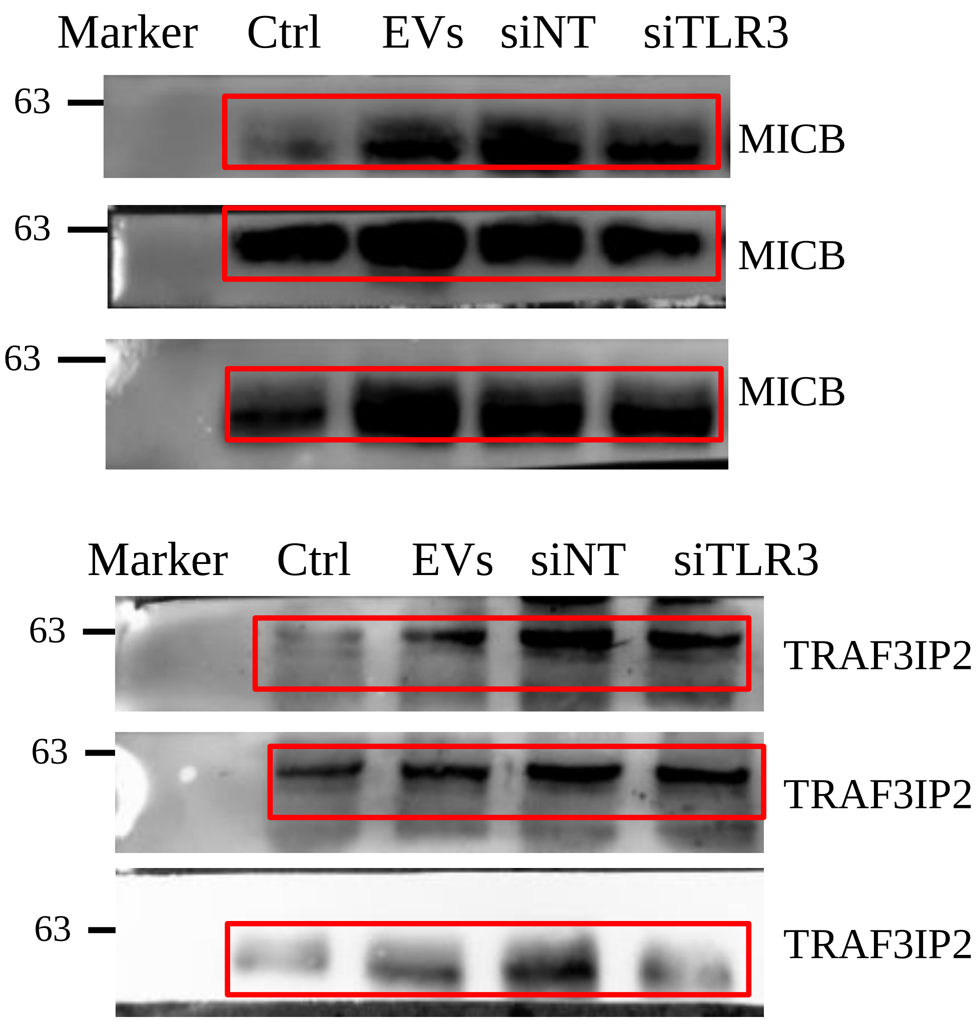

Fig 9D. The raw data of western blotting.
The boxed regions were shown in the article.

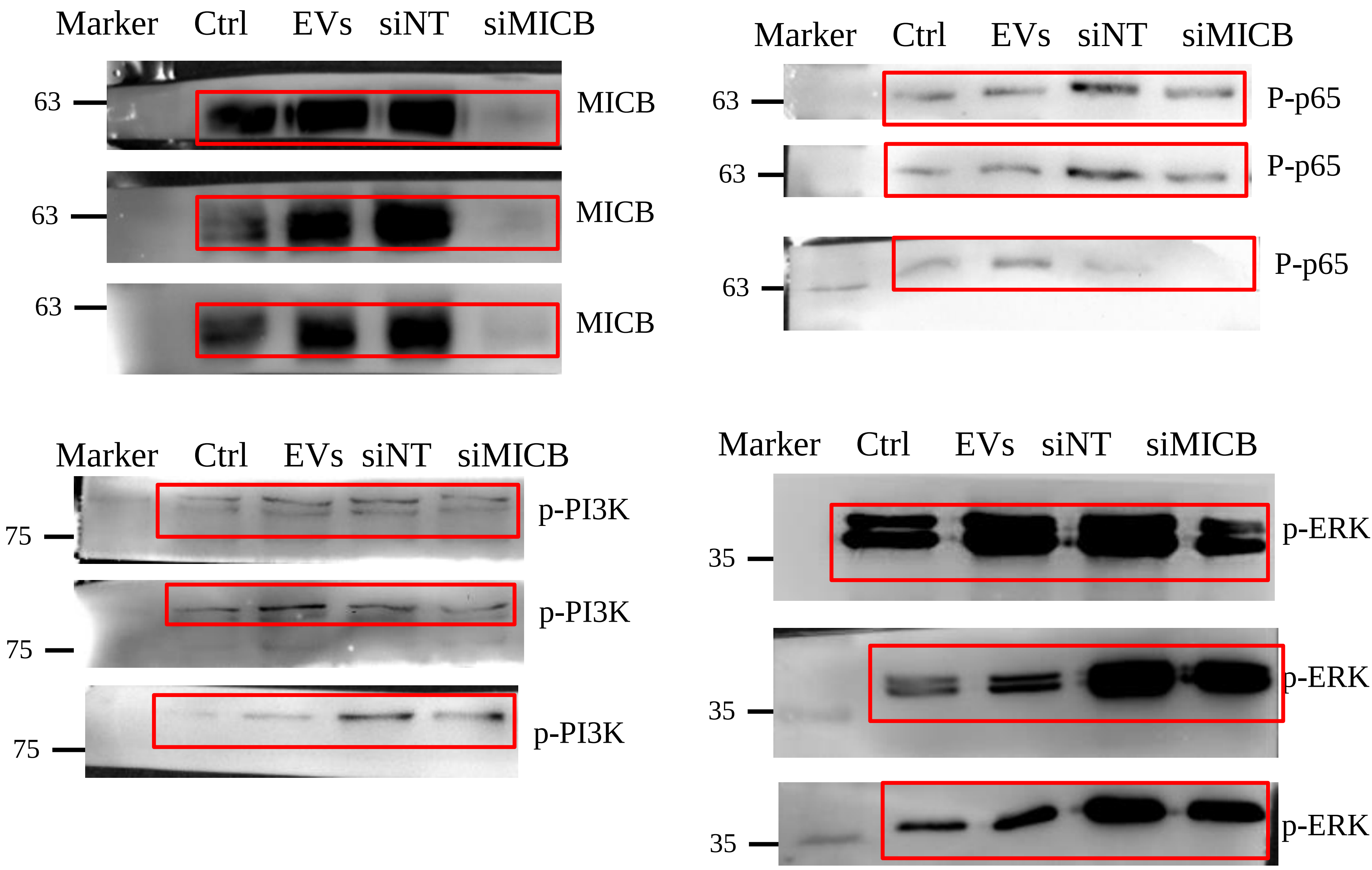

Fig 9E. The raw data of western blotting.
The boxed regions were shown in the article.

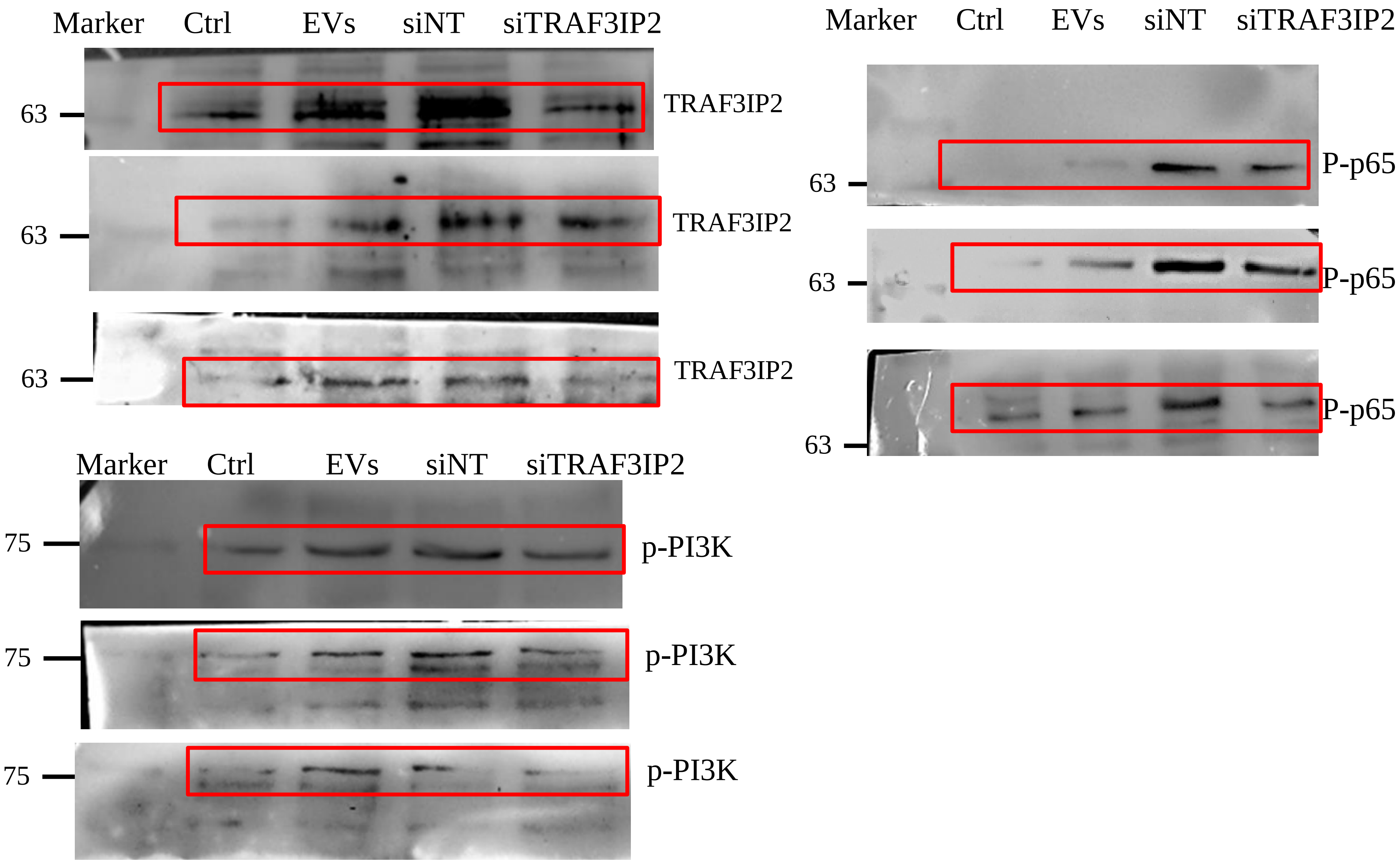

Fig 9E. The raw data of western blotting.
The boxed regions were shown in the article.

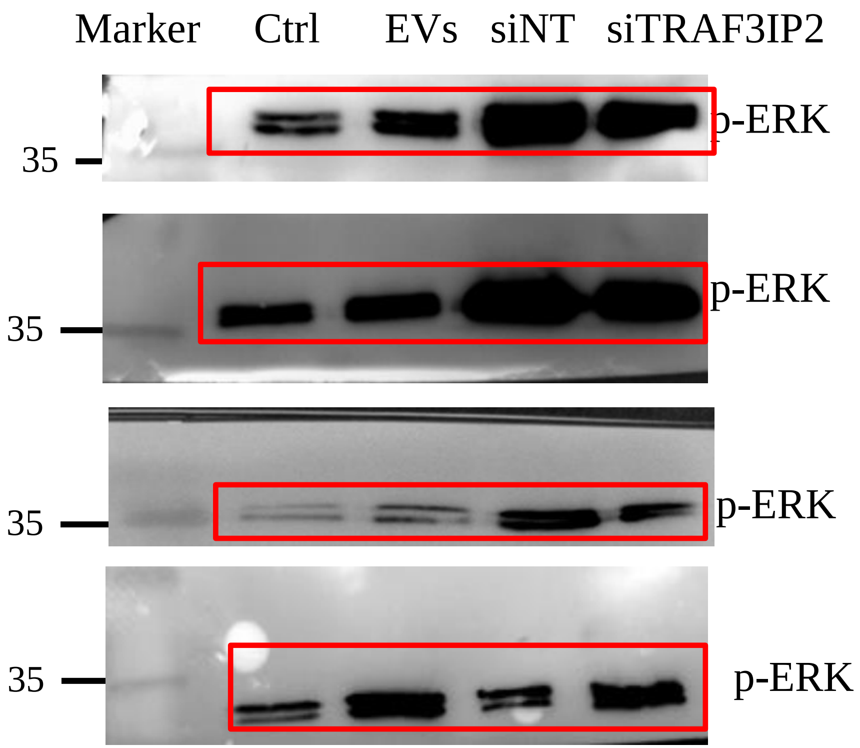

Fig 9E. The raw data of western blotting.
The boxed regions were shown in the article.
