# Supplementary material for: Trichomonas vaginalis extracellular vesicles activate the NLRP3 inflammasome and TLR3-mediated inflammatory cascades in host cells
Source: PLoS Pathog. 2025 Jun 2;21(6):e1013216. doi: 10.1371/journal.ppat.1013216 (PMC12157993; doi:10.1371/journal.ppat.1013216)
Supplement: Attachment — Submitted filename: Response to reviewers comments_Final.pdf [file ppat.1013216.s011.pdf]

Responses to reviewers' comments

Reviewers' Comments:

Reviewer's Responses to Questions

Part I - Summary

Please use this section to discuss strengths/weaknesses of study, novelty/significance, general execution and scholarship.

Reviewer #2: The authors have addressed the questions and comments previously given by this referee.

Response:

We thank the reviewer for taking the time to review our revised manuscript and giving us the positive feedback.

Reviewer #3: The authors used three strains of Trichomonas vaginalis to demonstrate that TLR3 is involved in the induction of NLRP3 inflammasomes by T. vaginalis in THP-1 cells. This revised manuscript has been reviewed by two reviewers, and the author has addressed all their questions. The manuscript has reached a fairly high level of completion. However, I hope the author can clarify a few remaining points:

Response:

We very appreciate the insightful comments and positive feedback raised by the reviewer. Please see below our detailed responses to the reviewer's comments.

Part II – Major Issues: Key Experiments Required for Acceptance

Please use this section to detail the key new experiments or modifications of existing experiments that should be absolutely required to validate study conclusions.

Generally, there should be no more than 3 such required experiments or major modifications for a "Major Revision" recommendation. If more than 3 experiments are necessary to validate the study conclusions, then you are encouraged to recommend "Reject".

Reviewer #2: None

Response:
We thank the reviewer for the positive feedback.

Reviewer #3: No experimental modifications are required.

Response:
We thank the reviewer for evaluating our manuscript and giving us the positive feedback.

Part III – Minor Issues: Editorial and Data Presentation Modifications
Please use this section for editorial suggestions as well as relatively minor modifications of existing data that would enhance clarity.

Reviewer #2: None

Response:
We appreciate the positive feedback raised by the reviewer.

Reviewer #3: 1. The results presented in Figure 4 indicate that TV-EVs activate the NLRP3 inflammasome and subsequently induce IL-1β secretion in THP-1 macrophages. Previous studies have confirmed that NLRP3 can activate caspase-1 by allowing ASC to bind to pro-caspase-1 [1]. This process triggers the activation of caspase-1 and the maturation and secretion of pro-inflammatory cytokines, such as interleukin-1β (IL-1β) and IL-18. However, this study only confirmed the secretion of IL-1β in THP-1 macrophages and did not investigate IL-18. The author should discuss and explain this in the discussion section.

Response:
We thank the reviewer for raising this point to our attention. As recommend by the reviewer, we have discussed this issue in the discussion section as follows. "Similarly, we found that TV-EVs could promote IL-1β secretion. Activation of the NLRP3 inflammasome is typically associated with the secretion of both IL-1β and IL-18 [1]. Although IL-18 is also processed by caspase-1, IL-1β is more commonly assessed as a representative cytokine of NLRP3 inflammasome activation [2, 3]. Hence, our analysis focused on IL-1β secretion in THP-1 macrophages treated with TV-EVs.

Future studies could investigate whether TV-EVs also promote IL-18 release, thereby providing a more comprehensive understanding of the TV-EV-induced immune response in host cells."

References:
1. Swanson, K.V., M. Deng, and J.P. Ting, The NLRP3 inflammasome: molecular activation and regulation to therapeutics. Nat Rev Immunol, 2019. 19(8): p. 477-489.
2. Cullen, S.P., et al., Diverse Activators of the NLRP3 Inflammasome Promote IL-1beta Secretion by Triggering Necrosis. Cell Rep, 2015. 11(10): p. 1535-48.
3. Minns, M.S., et al., NLRP3 selectively drives IL-1beta secretion by Pseudomonas aeruginosa infected neutrophils and regulates corneal disease severity. Nat Commun, 2023. 14(1): p. 5832.

2. Trichomonas vaginalis inflammasome activation induces macrophage inflammatory cell death by pyroptosis, which occurs through caspase-1 cleavage of the gasdermin D protein, leading to pore formation in the host cell membrane [2]. In this manuscript, the authors demonstrated that TV-EVs induce TLR3 overexpression, leading to the activation of the NF-κB/NLRP3 pathway in THP-1 macrophages. Would the use of TV-EVs induce apoptosis or pyroptosis in in vitro experiments? Please address this question in the discussion section.

1. Malik A, Kanneganti TD. Inflammasome activation and assembly at a glance. J Cell Sci. 2017;130(23):3955-63. doi: 10.1242/jcs.207365. PubMed PMID: 29196474; PubMed Central PMCID: PMCPMC5769591.
2. Riestra AM, Valderrama JA, Patras KA, Booth SD, Quek XY, Tsai CM, et al. Trichomonas vaginalis Induces NLRP3 Inflammasome Activation and Pyroptotic Cell Death in Human Macrophages. J Innate Immun. 2019;11(1):86-98. Epub 20181102. doi: 10.1159/000493585. PubMed PMID: 30391945; PubMed Central PMCID: PMCPMC6296884.

Response:

We thank the reviewer for raising this critical issue. As recommend by the reviewer, we have discussed this point in the discussion section as follows. "TV has been shown to induce mitochondria-dependent activation of caspases and phosphorylation of p38 MAPK, leading to apoptotic cell death in macrophages [1]. Additionally, TV has been reported to trigger caspase-1-mediated cleavage of gasdermin D, resulting in pyroptotic cell death in macrophages [2]. In our study, we demonstrated that TV-EVs activate the NLRP3 inflammasome and caspase-1 in THP-1 macrophages, leading to

cytotoxic effects in host cells upon prolonged exposure. These results suggest that TV-EVs may also induce pyroptosis in macrophages. Further investigations are warranted to elucidate the molecular mechanisms underlying host cell death in response to extended TV-EV exposure.

References:

1. Chang, J.H., et al., Apoptosis of macrophages induced by Trichomonas vaginalis through the phosphorylation of p38 mitogen-activated protein kinase that locates at downstream of mitochondria-dependent caspase activation. Int J Biochem Cell Biol, 2006. 38(4): p. 638-47.

2. Riestra, A.M., et al., Trichomonas vaginalis Induces NLRP3 Inflammasome Activation and Pyroptotic Cell Death in Human Macrophages. J Innate Immun, 2019. 11(1): p. 86-98.
