# Supplementary material for: Trichomonas vaginalis extracellular vesicles activate the NLRP3 inflammasome and TLR3-mediated inflammatory cascades in host cells
Source: PLoS Pathog. 2025 Jun 2;21(6):e1013216. doi: 10.1371/journal.ppat.1013216 (PMC12157993; doi:10.1371/journal.ppat.1013216)
Supplement: Attachment — Submitted filename: 20241018_Response to reviewers comments _Final.docx [file ppat.1013216.s010.docx]

**Responses to reviewers’ comments**

**Part I - Summary**
Please use this section to discuss strengths/weaknesses of study, novelty/significance, general execution and scholarship.

**Reviewer #1:** General comments: Three Trichomonas vaginalis strains were used, one of which was isolated from a patient. (2019) revealed that Trichomonas vaginalis induces the NLRP3 inflammasome in THP-1, and it was also reported that Tv-EV is internalized into ectocervical cells. TLR2, TLR3, TLR4 and TLR5 have been reported to be involved in the secretion of proinflammatory cytokines and chemokines from cervicovaginal mucosa. There is also a paper reporting proteomic analysis of EVs conducted by the Petricia group. However, in this paper, a total of three TVs were used, including Trichomonas vaginalis isolated from a patient, and this paper appears to be the first to reveal that TLR3 is involved in the induction of NLRP3 inflammasome in THP-1 cells by Tv. In addition, inflammasome-related experiments were conducted well, and additional proteomic data was used to identify key substances using western blot.

**Response:**

We would like to thank this reviewer for taking the time to examine our manuscript and bringing to our attention the lack of clarity of this manuscript. We have improved the critical points raised by the reviewer. Please find below the detailed point-by-point responses to the reviewer’s comments.

Points to improve: In this experiment, THP-1 cells and ectocervical cells were treated with Ev isolated from three strains of Trichomoniasis vaginalis, and the downstream signals of the inflammatory response within the cells were examined. However, the experiments on THP-1 cells and ectocervical cells were not conducted in parallel and in pairs, and in some experiments, only one cell was used, which overall feels disorganized. According to the authors' results, the micrographs appear to have been made using a general fluorescence microscope, and the resolution is low, so they do not accurately show the distribution and location of Ev, p65 NF-kB, etc. within the cell. And, some western results show that the expression of p65Nf-kB is too weak.

**Response:**

We thank the reviewer for bringing these critical issues to our attention, which significantly improve the quality of our manuscript. In the ectocervical cell (Ect) experiments (**Fig. 6** and **8**), we aimed to assess the activation of known inflammatory pathways (PI3K, AKT, NF-κB, etc.) and TLR3 expression in Ect treated with TV-EVs for various time intervals. Similarly, in the THP-1 experiments (**Fig. 5** and **7**), we aimed to examine the p-p65 NF-kB and TLR3 expression in THP-1 treated with TV-EVs for different time intervals. For these experiments, we used TV-EVs isolated from a single Tv strain. However, we still used TV-EVs from three different Tv strains for validation. For example, three different TV-EVs were employed in cytokine secretion assays after treatment with pathway inhibitors and the TLR3-targeting siRNA in Ect (**Fig. 6F** and **8D**) and in THP-1 cells (**Fig. 5D-E** and **7C**). In this way, we can alleviate the concerns about whether different TV-EVs might produce varying results.

Regarding the quality of the micrographs, we have thoroughly improved the fluorescent staining images **(Fig. 2A-B、4D、5C、6C** and **6E)** by utilizing confocal microscopy, providing clearer and high-resolution images. Additionally, we have improved the western blot results for p-p65 NF-κB expression **(Fig. 5A)**, enhancing the clarity of the blots. In the revised manuscript, we have replaced all the general fluorescent images and low-quality western blot results with high-quality images. The detailed responses addressing these points are shown in the section of Major Issues raised by the reviewer.

**Reviewer #2:** The authors reported an interesting array of results aiming to understand molecular mechanisms of Tv-derived EV mediated immune responses in human THP-1 macrophages and ectocervical cells. The study encompasses cell biological, immunological, and proteomic analyses to explain how Tv EVs manipulate host cell NF-κB/NLRP3 signaling pathways in macrophages and PI3K-mediated NF-κB, p38 MAPK and ERK pathways in Ect. Apart from experiments in vitro, the authors also demonstrated some of their findings in vivo. This paper provides significant contribution to our understanding of Tv EV-mediated pathogenesis and host immune response. However, there are some minor details I wish to be clarified.

**Response:**

We truly appreciate the positive feedback from the reviewer. We hope that we have responded appropriately to the concerns that have been made. Please find below the detailed point-to point responses to each comment as follows.

**Part II – Major Issues: Key Experiments Required for Acceptance**
Please use this section to detail the key new experiments or modifications of existing experiments that should be absolutely required to validate study conclusions.

Generally, there should be no more than 3 such required experiments or major modifications for a "Major Revision" recommendation. If more than 3 experiments are necessary to validate the study conclusions, then you are encouraged to recommend "Reject".

**Reviewer #1**: Fig. 2A.2B states that Tv exosomes are internalized in THP-1 cells or ectocervical cells. However, fluorescence microscopy images have low resolution and low magnification, making the cells appear small in size. Therefore, it does not accurately show evidence that Ev is internalized inside the host cell. Using confocal microscopy, Ev internalization can be observed more clearly.

**Response:**

We thank the reviewer for raising this constructive comment and giving us the opportunity to explain this issue. For the internalization assay (please see the Materials and Methods), TV-EVs were stained with PKH-67 and co-incubated with host cells for different time intervals. After co-incubation, the cells were washed with PBS three times to remove any uninternalized TV-EVs. This ensures that only the internalized TV-EVs within the host cells are visualized under the fluorescence microscope. In response to the reviewer’s concern regarding the low-resolution and magnification of the images, we have utilized confocal microscopy to improve the resolution and clarity of the images in the revised manuscript (**Fig. 2A** and **2B**).

Additionally, just as exosomes are stained, the cytoplasm of the host cell must also be stained. By additionally using a dye that can stain the cytoplasm, such as CtxB-Alex, you can clearly observe that Ev is internalized into the cytoplasm of the host cell. Additionally, an indication of the microscope magnification at which the observations were made is also required. It would be nice to have an enlarged photo showing the internalization of EV. In addition, since Ev internalization into cells occurs early, it is necessary to observe the EV processing time for less than 3 hours (e.g., 30 minutes, 1 hour, 2 hours, 3 hours, etc.) to show the changing pattern. It's the same.

**Response:**

We appreciate these constructive comments, which have greatly enhanced the quality of our manuscript. In this experiment, we aimed to validate the internalization of TV-EVs into host cells, rather than to focus on their precise localization within the cytoplasm. Therefore, we utilized CellMask™ plasma membrane staining to stain the plasma membrane and observe the internalization of TV-EVs into host cells. Additionally, as recommended by the reviewer, we have performed additional experiments to illustrate the time-dependent internalization pattern of TV-EVs (30 minutes, 1 hour, 2 hours, 3 hours, and 6 hours) (**Fig. 2A** and **2B**) using confocal microscopy.

We have revised the description regarding the internalization of TV-EVs in the results section as follows. “The fluorescent dye PKH67 was used to label the phospholipid bilayer-bound structures of TV-EVs, followed by incubation with THP-1 macrophages and Ect for different time intervals. The cells treated with PBS were served as a negative control. Immunofluorescence data showed that punctate signals of TV-EVs were detected in host cells after 1 hour of co-incubation and increased in a time-dependent manner (**Fig 2A-B**). After 3 hours of co-incubation, numerous punctate signals of TV-EVs were observed fusing with the plasma membranes of THP-1 macrophages and Ect.”

**Reviewer #2:** 1. Can the authors explain why only physical characterization of EVs amongst three Tv strains was conducted? A comparative proteomic or RNA profiling of EVs derived from three Tv isolates may provide better insights into the differences in the cytokine/chemokine responses elicited in human cells. If this analysis is performed, it may significantly improve the discussion which is currently limited to generalization as Tv EVs and not to specific proteins/RNAs directly involved in affecting host pathways.

**Response:**

We thank the reviewer for raising this constructive comment. In the study, we aim to identify the molecular mechanisms of inflammation induced by TV-EVs and identify novel proteins modulating the immune response in Ects. Hence, we mainly focused on the host immune response stimulated with TV-EVs isolated from various strains. We used three different TV-derived EVs and want to establish the common profiles of inflammatory cytokines, inflammatory pathways, and host proteomes to convince that the inflammatory cascade induced by different TV-EVs is a common phenomenon in host cells. In the first part of this study **(Fig. 1)**, we need to confirm that the isolation of TV-EVs from three strains is successful. Hence, we characterized the TV-EVs from three stains using NTA and TEM. However, in this study, we do not aim to identify the specific proteins/RNAs inside the TV-EVs from different strains, because we want to first ensure the host inflammatory responses induced by different TV-EVs are common, as previously described, but not specific to any TV-derived EVs or to EV proteins.

Regarding the reviewer's comment for proteomic comparison of EVs isolated from different strains, another study in our group is currently ongoing. We have already proved that TV-EVs derived from metronidazole-resistant (MTZ-R) strains can deliver MTZ resistance to metronidazole-sensitive (MTZ-S) strains (Unpublished data). Hence, we performed proteomic analysis of TV-EVs from three MTZ-R and three MTZ-S strains, trying to identify the specific proteins inside TV-EVs from MTZ-R strains that mediate the MTZ resistance. In this case, we focused on the drug resistance mechanism mediated by specific proteins in TV-EVs.

2. The authors should also highlight and provide plausible reasons when there is differences in the responses when EVs from three different Tv strains is used.

**Response:**

We appreciate the constructive comment raised by the reviewer. We had previously mentioned this point in the discussion section of the original manuscript. To address the reviewer's suggestion further, we have added more discussion to the revised manuscript as follows, with the new additions highlighted in red.

“Notably, we observed a significant release of CXCL1 in THP-1 macrophages and Ect stimulated with TV-EVs (**Fig. 3D** and **3F**), especially those derived from the clinical strain (NDMC5). This observation suggests that TV-EVs isolated from different strains can induce varying intensities of host immune responses, potentially due to differences in their protein compositions. A previous study reported that exosome-like vesicles isolated from the TV GT-21 strain induces higher expression of IL-10, IL-6 and TNF-α in RAW264.7 macrophages [1], supporting that EVs from different TV strains may trigger distinct immune responses.”

“We observed that NDMC5 EVs did not significantly alter IL-8 secretion in THP-1 macrophages pretreated with a TLR3 inhibitor or si-TLR3, suggesting that TLR3 may not be involved in the regulation of NDMC5 EV-induced IL-8 secretion. This finding further supports the idea that differences in the protein composition of EVs may activate distinct signaling pathways, leading to varied regulation of inflammatory cytokine secretion in host cells.”

Reference

1. Olmos-Ortiz LM, Barajas-Mendiola MA, Barrios-Rodiles M, Castellano LE, Arias-Negrete S, Avila EE, et al. *Trichomonas vaginalis* exosome-like vesicles modify the cytokine profile and reduce inflammation in parasite-infected mice. Parasite Immunol. 2017;39(6).

3. Why are some experiments done not in parallel? Is there a reason why proteomic analysis after co-incubation of Tv EVs in Ect only, and not in macrophage was conducted?

**Response:**

A previous study has demonstrated that TV induces NLRP3 inflammasome activation in human macrophages [1]. Hence, our study primarily aimed to investigate whether TV-derived EVs could similarly induce NLRP3 inflammasome activation in THP-1 macrophages. Our work demonstrates for the first time a contact-independent mechanism by which TV-EVs activate the NLRP3 inflammasome in THP-1 macrophages.

To establish and maintain infection, TV must adhere to epithelial cells in the vaginal ecosystem, a process that is poorly understood. Current knowledge regarding the molecular mechanisms by which TV triggers inflammation in vaginal epithelial cells (VECs) or ectocervical cells (Ects) is limited. The first study on TV exosomes demonstrated that these EVs can modulate host cell immune responses [2]; however, the underlying molecular mechanisms are still unknown. Hence, in this study, we aim to investigate not only the known inflammatory pathways (PI3K, NF-κb, p38 MAPK and ERK) in Ects stimulated with TV-EVs, but also to identify novel proteins or pathways through proteomic analysis.

References:

1. Riestra AM, Valderrama JA, Patras KA, Booth SD, Quek XY, Tsai CM, et al. Trichomonas vaginalis Induces NLRP3 Inflammasome Activation and Pyroptotic Cell Death in Human Macrophages. J Innate Immun. 2019;11(1):86-98.

2. Twu O, de Miguel N, Lustig G, Stevens GC, Vashisht AA, Wohlschlegel JA, et al. Trichomonas vaginalis exosomes deliver cargo to host cells and mediate hostratioparasite interactions. PLoS Pathog. 2013;9(7)

**Part III – Minor Issues: Editorial and Data Presentation Modifications**
Please use this section for editorial suggestions as well as relatively minor modifications of existing data that would enhance clarity.

**Reviewer #1**: Fig. 2C, This is an electron micrograph of vesicles internalized into cells. However, to be honest, I'm not sure if anything the authors show is clearly an Exosome. This is because macrophages can undergo exocytosis and secrete exosomes in response to Tv-EV, and cells are known to secrete exosomes. Electron micrographs appear to be mixed. Therefore, it would be good if the authors could more clearly indicate the exosomes they observed using arrows, etc. Also, the scale bar of the electron microscope photo needs to be corrected from 1um to 1μm.

**Response:**

We thank the reviewer for bringing this point to our attention. As recommended by the reviewer, we have made the corrections in the revised manuscript. In **Fig. 2C** and **2D**, the arrows have been added to clearly indicate the TV-EVs. Additionally, the scale bar has been corrected from “1um” to “1 μm”.

Fig. 5, When treated with Ev, the p-p65 NF-kB band in THP-1 cells is too faint. And, Fig. The authors claim that NF-kB p65 moved to the nucleus in 5C, but this is difficult to confirm in micrographs. It can be clearly seen only when taken with a confocal microscope. Comparing the merged pictures, it appears that NF-kB moved to the nucleus more in the control treated group than in the TV-EV treated group. To assert the movement of NF-kB to the nucleus, it is necessary to separate the cytosol and nuclear fractions and confirm them by Western blot.

**Response:**

We thank the reviewer for highlighting these issues, which have significantly improved the quality of our manuscript. Regarding the expression of p-p65 NF-κB in THP-1 cells, we have improved the quality of the western blot images and clearly demonstrate that TV-EVs treatment enhances the expression p-p65 in THP-1 cells (**Fig. 5A**).

Additionally, as recommended by the reviewer, we have performed confocal microscopy to assess the localization of NF-κB. Our results indicate a clear trend of NF-κB translocation from the cytosol to the nucleus in THP-1 macrophages stimulated with TV-EVs **(Fig. 5C)**.

Furthermore, we have performed additional experiments to validate the translocation of NF-κB by separating the cytosol and nuclear fractions. We have updated the description regarding the expression of NF-κB in these fractions in the revised manuscript as follows. “To further confirm this observation, nuclear and cytoplasmic extracts from THP-1 macrophages treated with TV-EVs for 4 hours were analyzed, revealing that the expression of NF-κB p65 was upregulated in the nuclear fraction of the TV-EV-treated group (**S2 Fig**).”

Fig. 6C and D, high-resolution photographs are required, and the movement of NF-kB to the nucleus can be observed only through confocal microscopy rather than fluorescence microscopy.

**Response:**

As recommended by the reviewer, we have performed confocal microscopy to enhance the clarity of the images **(Fig. 6C** and **6E)** in the revised manuscript. These images clearly indicate the significant translocation of NF-κB into the nucleus in Ects treated with TV-EVs. Additionally, Ect pretreated with the PI3K inhibitor wortmannin and then co-cultured with TV-EVs showed a marked reduction in NF-κB p65 nuclear translocation, suggesting that TV-EVs activate the NF-κB p65 pathway through PI3K signaling.

Fig. 8, TLRs (1, 2, 4, 5, 6, 10, 11) are mainly found on the cell surface, and TLRs (3, 7, 8, 9) are known to exist on the intracellular endosomal membrane. TLR3 is said to be found on the cell surface of immune cells. It is difficult to identify the distribution and location within the cell from the fluorescence microscope images shown by the authors. If we are simply showing that TLR3 expression is increased, I think Figures 8A and 8C are sufficient.

**Response:**

We agree with the reviewer’s comment. As recommended by the reviewer, we have removed the fluorescence microscope images related to the expression of TLR3 in Ect (**Fig. 8B)**. A similar issue was also identified in THP-1 cells treated with TV-EVs. Hence, the TLR3 localization images of THP-1 treated with TV-EVs have also been removed (**Fig. 7B)** in the revised manuscript.

This paper shows for the first time that Tv-Ev alone is sufficient to activate the NLRP3 inflammasome in THP-1 cells. However, most experiments on inflammasome were conducted only on THP-1 cells, and inflammatory cytokine production by Tv-Ev and proteomic analysis as the last data were performed on ectocervical cells.
The relationship with the inflammasome was not proven in ectocervical cells, and the results in THP-1 cells and ectocervical cells were described unclearly in the review section. For a more complete paper, it would be better to publish the two contents separately.

**Response:**

We appreciate this constructive comment that is similar to a concern raised by another reviewer. A previous study demonstrated that TV induces NLRP3 inflammasome activation in human macrophages. Hence, our study primarily aimed to investigate whether TV-derived EVs could also induce NLRP3 inflammasome activation in THP-1 macrophages. Additionally, we assessed the production of inflammatory cytokines induced by TV-EVs in both THP-1 macrophages and Ect **(Fig. 3)**. Regarding the inflammasome study in Ect, we previously tested whether TV can activate the NLRP3 inflammasome, but the results showed that NLRP3 expression was not significantly upregulated in response to TV infection (data not shown). Hence, inflammasome activation in Ect was not a focus in this study.

Given the limited understanding of the molecular mechanisms by which TV triggers inflammation in vaginal epithelial cells (VECs) or Ect, we have placed greater emphasis on this aspect of this study. In addition to examining the known inflammatory pathways (PI3K, NF-κb, p38 MAPK and ERK) in Ect stimulated with TV-EVs, we also aimed to identify novel proteins or pathways involved in the TV-EV-induced inflammatory response through proteomic analysis.

**Reviewer #2:** 1. In Results, page 11, I do not clearly understand why the authors decide to use CD63 to test their Tv EVs. Is there Tv protein homologous to CD63? If so, please indicate in the manuscript.

**Response:**

We thank the reviewer for raising this point and giving us the opportunity to clarify. Since horse serum is required for the optimal culture medium of TV, we first assessed whether EVs could be successfully isolated from the optimal serum-containing culture medium (**Fig. 1B**), which contain both mammalian EVs and TV-EVs. As CD63 is a well-known marker for mammalian EVs, its detection in the EV fraction was anticipated in the presence of serum. Our results confirmed this expectation, showing CD63 expression in the serum-containing medium, thereby validating the accuracy of our EV isolation procedure. To exclude the influence of mammalian EVs, we subsequently isolated TV-derived EVs using serum-free culture medium. Under these conditions, CD63 expression was not detected, confirming the absence of mammalian EVs in our experimental system **(Fig. 1B).**

2. In Results, page 13, the authors assessed the effect of Tv EVs on proliferation of host cells. They showed declined proliferation of THP-1 macrophages after 24 h incubation with Tv EVs presented in supplementary figure 1A-D. These phenotypes are also important but sufficient discussion was not included. What could have caused this phenomenon?

**Response:**

We appreciate the reviewer for raising this issue. In this study, the impact of TV-EVs on the viability of THP-1 macrophages and Ect was assessed. This experiment aimed to determine the optimal time points for co-culturing host cells with TV-EVs. After 12 hours of co-incubation, the cell morphology became irregular and shrinking. A significant decrease in cell survival was observed after 24 hours of co-incubation. Based on these observations, we investigated the inflammatory mechanisms in host cells treated with TV-EVs within 12 hours in the subsequent experiments.

The reviewer raised a valuable point for future study why TV-EVs cause significant cell death after prolonged incubation. We propose that TV-EVs may contain cytotoxic molecules that may contribute to host cell death after prolonged co-incubation. Future studies could explore this by conducting transcriptomic and proteomic analyses of TV-EVs to identify specific molecules responsible for inducing cytotoxicity in host cells.

Minor grammatical errors:

1. Abstract: ectocervial to ectocervical

2. Results, page 11: obviously to obvious

**Response:**

We thank the reviewer for his/her careful reading. We have corrected “ectocervial” into “ectocervical” and “obviously” into “obvious”.
